# Microwave-Assisted Rapid Extraction of Oleuropein from Olive Leaf By-Product and Processing into Oleuropein@Zeolite Nanohybrids for Antioxidant Food Applications (Fortified Salt and Active Gelatin Films)

**DOI:** 10.3390/molecules31111833

**Published:** 2026-05-26

**Authors:** Achilleas Kechagias, Andreas Giannakas, Panagiotis Stathopoulos, Maria Xenaki, Areti A. Leontiou, Anna Kopsacheili, Nikolaos Chalmpes, Emmanuel P. Giannelis, Constantinos E. Salmas, Charalampos Proestos, Aris E. Giannakas

**Affiliations:** 1 Department of Food Science and Technology, School of Agricultural Sciences, University of Patras, 2 G. Seferi Str., 30100 Agrinio, Greece; up1110842@upatras.gr (A.K.); andgiannakas@upatras.gr (A.G.); aleontiu@upatras.gr (A.A.L.); 2 Division of Pharmacognosy and Natural Products Chemistry, Department of Pharmacy, National and Kapodistrian University of Athens, 15771 Athens, Greece; stathopan@pharm.uoa.gr (P.S.); maxenaki@pharm.uoa.gr (M.X.); 3 PharmaGnose S.A., Papathanasiou 24, 34100 Chalkida, Greece; 4 Laboratory of Food Chemistry, Department of Chemistry, National and Kapodistrian University of Athens Zografou, 15771 Athens, Greece; akopsacheili@chem.uoa.gr; 5 Department of Materials Science and Engineering, Cornell University, Ithaca, NY 14850, USA; nc427@cornell.edu (N.C.); epg2@cornell.edu (E.P.G.); 6 Department of Material Science and Engineering, University of Ioannina, 45110 Ioannina, Greece; ksalmas@uoi.gr

**Keywords:** olive leaf by-product, microwave-assisted extraction, oleuropein, natural zeolite, active gelatin films, circular bioeconomy

## Abstract

Olive leaves are an abundant agro-industrial by-product rich in oleuropein, yet they remain largely underutilized. The objective of this study is to a) develop a green microwave-assisted extraction (MAE) method for an oleuropein-rich extract, b) encapsulate it into edible natural zeolite to form OLE@NZ nanohybrids, and, c) evaluate their application in fortified salt and active gelatin films. MAE using only water at 96 °C for 5 min yielded a dry extract with 25.4% (*w*/*w*) oleuropein and a total phenolic content of 781 mg GAE/100 mL. The extract was successfully adsorbed onto clinoptilolite-type zeolite and the resulting nanohybrids showed strong antioxidant activity (EC_50,DPPH_ = 2.74 mg, TPC = 426 mg GAE/g). A fortified salt containing 5% *w*/*w* OLE@NZ fully preserved the nanohybrid’s antioxidant activity. Extruded gelatin films incorporating 5–15% OLE@NZ exhibited a concentration-dependent increase in antioxidant activity (up to 14-fold higher than the blank film), together with a 5- to 7-fold enhancement, while maintaining good mechanical properties. The total phenolic content of the films correlated linearly with nanohybrid loading, with phenolic recovery of 68% both at 5 and 10% loading and 58% at 15%). Overall, these findings demonstrate that MAE is a rapid, and environ-mentally friendly approach for obtaining oleuropein-rich olive leaf extract (OLE), while OLE@NZ nanohybrids provide effective antioxidant additives for functional salt formulations and active gelatin films, supporting a circular bioeconomy strategy.

## 1. Introduction

The valorization of food and agricultural by-products has emerged as a cornerstone of the circular bioeconomy, addressing both environmental sustainability and resource efficiency [1,2,3,4,5,6]. Among the most abundant yet underutilized biomass is olive leaves (*Olea europaea* L.), which are produced in massive quantities during olive cultivation and olive oil production. Commonly treated as waste, these leaves are often burned or disposed in landfills, contributing to environmental burden. Nevertheless, olive oils constitute a rich source of high-value bioactive compounds, and their exploitation represents a compelling economic and environmental opportunity [7,8,9,10,11,12].

Critical parameters influencing the bioactive composition of olive leaves extracts include the drying method and the extraction technique, applied to the plant material. The simplest drying approach is ambient air-drying; however, this method is associated with prolonged drying times and the production of extracts with reduced phenolic content, mainly due to enzymatic reactions occurring under ambient conditions [13]. An alternative low-cost approach is oven drying. Nevertheless, this method presents notable drawbacks, as high temperatures may lead to the degradation of heat-sensitive compounds present in olive leaves, whereas at moderate temperatures (40–50 °C), enzymatic reactions are still promoted, including the hydrolysis of Oleuropein into simpler phenolic derivatives [14]. Furthermore, based on previous work, freeze-drying results in extracts with reduced oleuropein content, due to enzymatic biotransformation of oleuropein into oleacein under freeze-drying (FD) conditions [15]. In contrast, microwave (MW) drying has been proposed as the most effective method for producing oleuropein-rich extracts from olive leaves [15]. During MW drying, high-intensity microwave irradiation is absorbed by the intrinsic moisture of the plant matrix, increasing the kinetic energy of water molecules and generating high-energy steam within cellular structures. This leads to disruption of cellular compartments and rapid dehydration of the plant material. Notably, the temperature during MW drying is sufficiently controlled to prevent damage to critical quality parameters, The short duration of the MW drying process combined with minimal energy losses due to direct microwave absorption by intrinsic water without the need for an external medium, makes this technique highly efficient [16]. Moreover, based on previous findings, during microwave drying of olive leaves, the enzymes have lost their activity, leaving the oleuropein molecule unaffected [15]. This explains why olive leaves dried using MW treatment exhibit the highest oleuropein content compared to other drying methods.

Another critical aspect for the more effective valorization of olive leaves is the development of green, rapid, and efficient extraction processes that eliminate the use of toxic organic solvents. Conventional solid–liquid extraction methods are often time-consuming, energy-intensive, and rely on petrochemical solvents, conflicting with green chemistry approaches [17,18,19,20]. In this context, microwave-assisted extraction (MAE) has emerged as an attractive alternative. MAE leverages volumetric heating to disrupt plant cell walls, thereby enhancing mass transfer and significantly reducing extraction time (minutes rather than hours) as well as solvent consumption. When performed using only water or food-grade solvents, MAE is particularly suitable for the production of food-compatible extracts [21,22,23,24,25,26,27,28].

Among the myriad polyphenols found in olive leaves, oleuropein is the most abundant and biologically significant. This secoiridoid glycoside is responsible for many of the health-promoting properties attributed to olive products, including potent antioxidant, anti-inflammatory, antimicrobial, and cardioprotective activities [29,30]. The efficient recovery of oleuropein in its native, undegraded form is therefore highly desirable. However, the direct application of oleuropein in food systems is often limited by its bitter taste, chemical instability (susceptibility to oxidation and hydrolysis), and potential for uncontrolled release [31,32,33,34,35,36]. A rapid, green MAE protocol that yields a stable, oleuropein-rich extract is thus a prerequisite for further functionalization.

To address these limitations, this study introduces a novel strategy: the development of edible nanohybrids composed of oleuropein and natural edible zeolite. Zeolites are microporous aluminosilicate minerals with well-documented cation-exchange capacity, high specific surface area, and a generally recognized as safe (GRAS) status for specific food applications [37,38]. The encapsulation or adsorption of oleuropein into the zeolite framework can potentially mask its bitterness, protect it from degradation during processing and storage, and enable a controlled release profile. Furthermore, the natural zeolite (NZ) itself may contribute beneficial properties, such as a modulating effect on mineral content or as a nanostructuring agent. To date, the formation of oleuropein@zeolite (OLE@NZ) nanohybrids using a green extract obtained by MAE has not been reported.

Finally, this work translates the laboratory-scale nanohybrids into two concrete, industrially relevant food applications. First, oleuropein- and zeolite-fortified salt is proposed as a novel functional seasoning. Salt (NaCl) is a universal food matrix; fortifying it with a natural antioxidant and a mineral carrier could provide a simple vehicle for delivering bioactive compounds while potentially mitigating the oxidative stress associated with high-sodium diets [38,39]. Second, the nanohybrids are incorporated into active gelatin films. Gelatin (Gel), a biodegradable and edible biopolymer, is an excellent film-forming material. The incorporation of OLE@NZ nanohybrids is expected to confer active packaging functionalities, such as antioxidant and antimicrobial activity, thereby extending the shelf life of perishable foods [40,41,42].

Thus, the main objectives of this study are: (i) to optimize a rapid, solvent-free micro-wave-assisted extraction method for an oleuropein-rich extract from olive leaf by-products; (ii) to fabricate and characterize novel edible OLE@NZ nanohybrids; and, (iii) to evaluate their performance in two prototype food systems, namely fortified salt and active gelatin films. We hypothesize that the zeolite nanocarrier will enhance the stability and functionality of the olive leaf extract, leading to improved antioxidant and antimicrobial performance in both applications.

## 2. Results

### 2.1. Characterization of the Microwave-Assisted OLE

#### 2.1.1. ATR-FTIR Analysis of OLE

**Figure 1:**
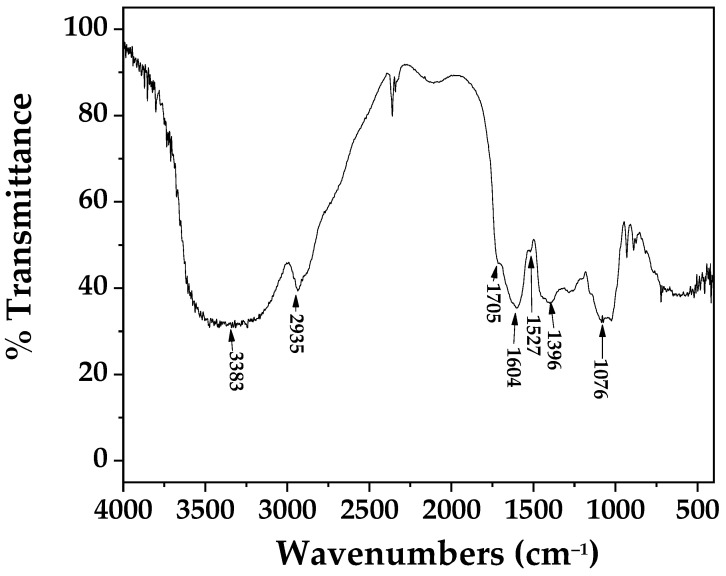
ATR-FTIR spectra of the lyophilized OLE powder.

The ATR-FTIR spectrum of the lyophilized OLE powder is shown in Figure 1 (spectrum 2).

The spectrum reveals the characteristic absorption bands of polyphenolic compounds: a broad O–H stretching envelope at ~3200–3400 cm^−1^, C–H stretches at ~2935 cm^−1^, a carbonyl (C=O) band at ~1705 cm^−1^, aromatic C=C vibrations at ~1604 and 1527 cm^−1^, and a C–O–C stretching band at ~1236 cm^−1^. These features are consistent with the presence of intact oleuropein and related secoiridoids, as previously reported [43,44]. The freeze-drying process did not alter the chemical structure, as the lyophilized material retains all characteristic bands.

#### 2.1.2. Yield and Oleuropein Content of the OLE-HPLC DAD and HPLC-MS Analysis

**Figure 2:**
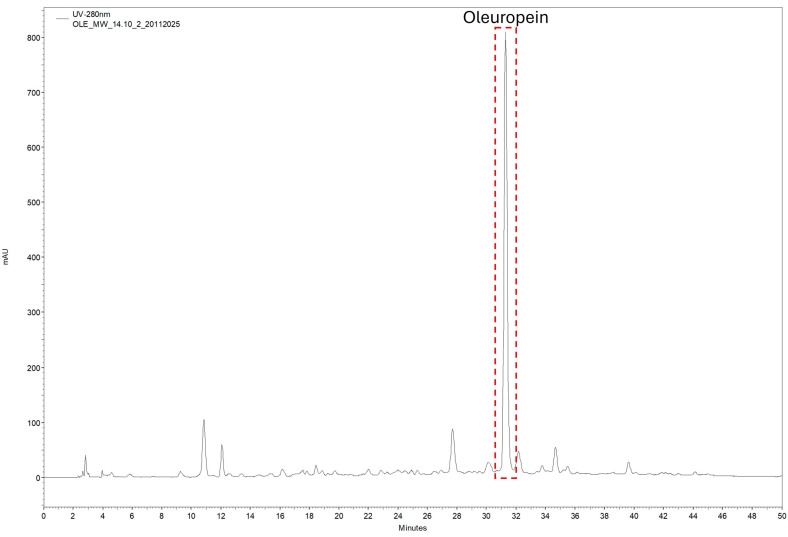
RP-HPLC-DAD chromatogram of the analyzed OLE recorded at 280 nm.

Lyophilization of the OLE obtained by MAE gave a dry extract yield of 0.551 g per 100 mL of the original aqueous extract. Quantitative HPLC-DAD analysis revealed an oleuropein content of 140.13 mg per 100 mL of extract. On a dry weight basis (using the dry extract yield of 0.551 g per 100 mL), this corresponds to 254 mg oleuropein per g of dry extract, or approximately 25.4% *w*/*w*. Figure 2 shows the RP-HPLC-DAD chromatogram of the OLE recorded at 280 nm. Oleuropein appears as the dominant peak at a retention time of 31.6 min.

The HPLC-MS analysis allowed the identification of 16 bioactive compounds in the OLE. The Retention times, Calculated mass (*m*/*z*), Proposed compounds, and Molecular Formula of identified compounds are summarized in Table 1.

**Figure 3:**
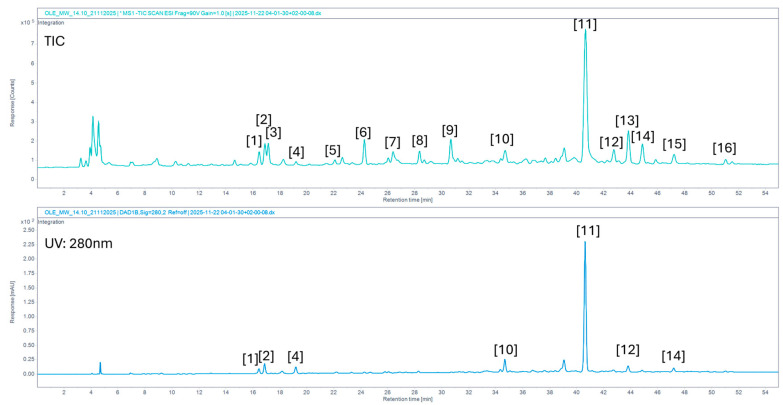
HPLC-PDA-MSQ chromatograms of the analyzed sample: (**upper**) total ion current (TIC) full-scan chromatogram; (**lower**) chromatogram recorded at 280 nm. The main peaks are labeled.

Figure 3 presents the HPLC-PDA-MSQ chromatograms: the upper trace is the total ion current (TIC) full-scan chromatogram, and the lower trace is the chromatogram recorded at 280 nm. The main peaks are labeled.

**Figure 4:**
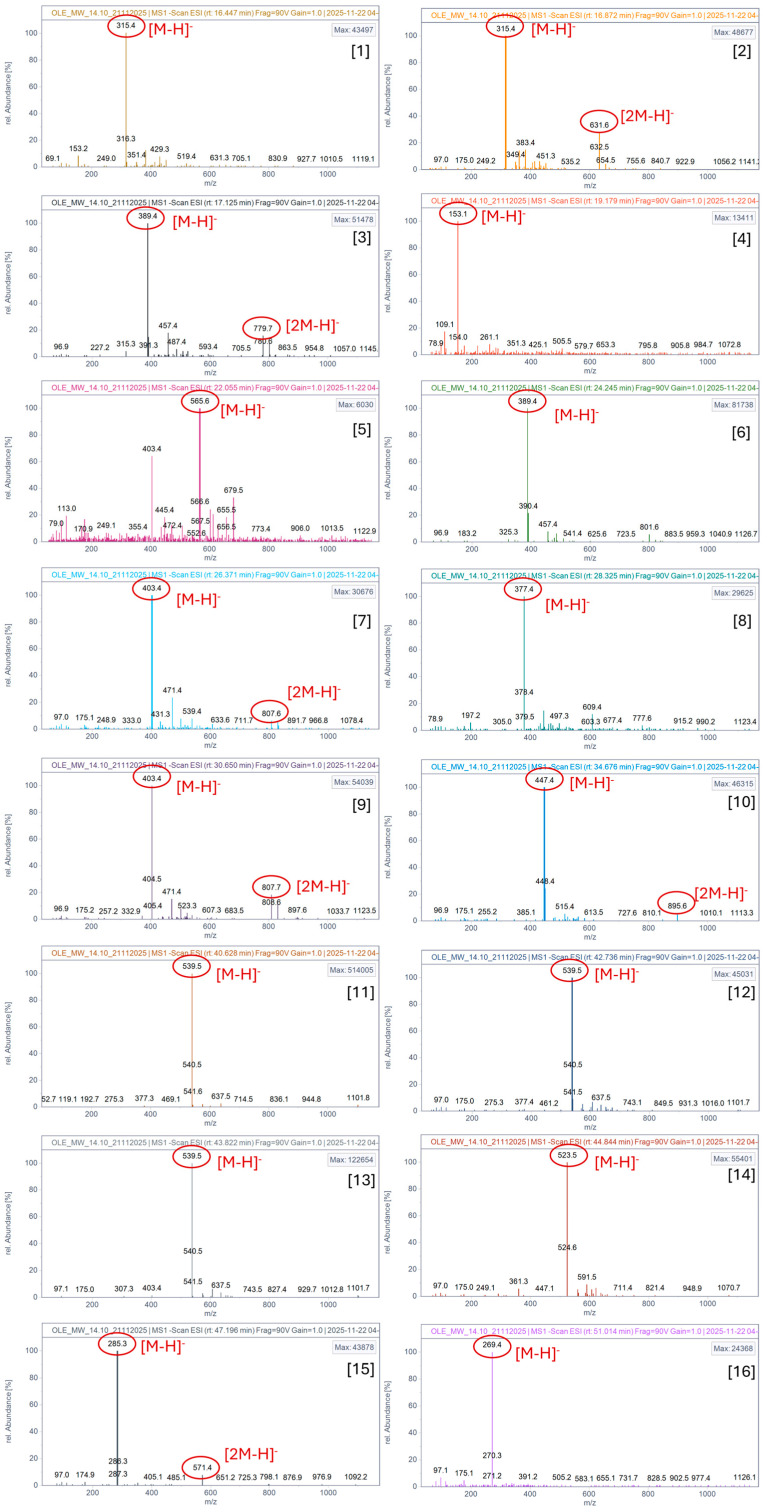
Mass spectra of the identified peaks.

Figure 4 shows the mass spectra of the identified peaks.

The most abundant compound was oleuropein (Rt = 40.628 min), confirming that the MAE efficiently recovered this major secoiridoid. Other significant peaks corresponded to oleuropein derivatives (oleuropein isomer, oleuroside), ligstroside, luteolin-7-O-glucoside, and simple phenolics such as hydroxytyrosol and its glucosides. The presence of elenolic acid derivatives and secoiridoid glucosides indicates a rich polyphenolic profile typical of *Olea europaea* leaves. Flavonoids (luteolin, apigenin) were also detected, contributing to the overall bioactivity of the extract.

The oleuropein concentration (140.13 mg/100 mL of extract) and the dry extract yield (0.551 g/100 mL) correspond to an oleuropein content of approximately 25.4% (*w*/*w*) in the lyophilized OLE. This value compares favorably with previously reported MAE protocols for olive leaves. For example, Şahin et al. (2017) obtained an oleuropein yield of 42.4 mg/g dry leaf using MAE with ethanol: water (80:20) at 80 °C for 10 min [27]. Using pure water as the solvent (as in our study) typically yields slightly lower but still competitive values; Rahmanian et al. (2015) reported 36 mg/g dry leaf under water-based MAE [45]. Assuming a solid-to-liquid ratio of approximately 1:20 (common for olive leaf MAE), our value translates to roughly 25 mg oleuropein per gram of dry leaves, which is well within the range (10–70 mg/g) reported for the Greek Koroneiki variety by Lafka et al. (2011) [46,47].

The detection of 16 phenolic compounds by HPLC-MS is consistent with the known phytochemical profile of olive leaves. Kontogianni et al. (2013) also identified oleuropein as the dominant compound in Greek olive varieties, accompanied by hydroxytyrosol, luteolin-7-O-glucoside, and oleuroside [48]. Importantly, the presence of intact oleuropein together with only modest amounts of its hydrolysis product hydroxytyrosol indicates that our mild MAE conditions (96 °C, 5 min) did not cause extensive thermal degradation. This is advantageous because oleuropein itself exhibits distinct bioactivities (e.g., antimicrobial, anti-inflammatory) compared to its aglycone [49,50]. The enzymatic degradation of Oleuropein is not favored due to the microwave (MW) drying method applied to the olive leaves. Microwave drying effectively inactivates endogenous leaf enzymes, thereby preventing biotransformation and hydrolytic reactions of oleuropein, during the extraction process, as previously described [15]. The identification of ligstroside, secologanoside, and secoxyloganin further confirms the typical secoiridoid profile of olive leaves, as reviewed by Soler-Rivas et al. (2000) [51].

Our results are also in agreement with Talhaoui et al. (2015), who reported that flavone glycosides (luteolin-7-O-glucoside) and aglycones (luteolin, apigenin) are characteristic markers of OLE and contribute significantly to antioxidant capacity [52]. Compared to conventional solid–liquid extraction (e.g., maceration for 24 h), which typically yields 50–80 mg oleuropein per 100 mL of extract [53], our MAE protocol provided almost double the concentration in only 5 min of active extraction time. Moreover, the use of deionized water as the sole extraction solvent avoids the need for organic solvents and subsequent solvent removal steps, making the extract directly suitable for food applications [17].

Finally, the high oleuropein content and the preservation of the native secoiridoid structure can be attributed to three key factors: (i) in the olive leaves drying technique, as previously described [15], and (ii) the controlled extraction temperature of 96 °C, which is below the threshold (110 °C) where thermal degradation of oleuropein becomes pronounced [54]. Collectively, these results demonstrate that the developed MAE protocol is rapid, green, and efficient for producing an oleuropein-rich OLE suitable for further nanohybrid formation and food applications.

#### 2.1.3. Surface Morphology of Freeze Fried OLE

**Figure 5:**
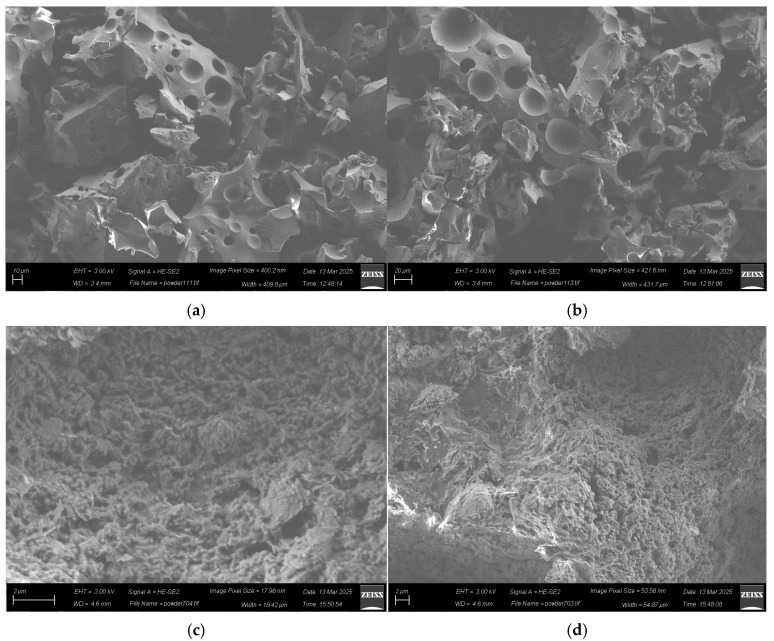
HR-SEM images of (**a**,**b**) freeze-dried OLE at two magnifications (scale bars as indicated), (**c**) natural zeolite (NZ), and (**d**) OLE@NZ nanohybrid.

The surface morphology of the lyophilized OLE was examined by high-resolution scanning electron microscopy (HR-SEM). Figure 5 presents representative micrographs at two magnifications.

The freeze-dried OLE exhibits a highly porous, sponge-like three-dimensional network with irregular, interconnected cavities. This characteristic morphology arises from the lyophilization process: the aqueous OLE is first frozen, forming ice crystals that act as a template. Subsequent sublimation of the ice under vacuum removes the water, leaving behind a porous scaffold composed of the concentrated polyphenolic matrix [55,56]. The pore walls appear thin and continuous, indicating that the dissolved solids (primarily oleuropein and other phenolics) were evenly distributed in the frozen state and did not collapse during drying.

No crystalline or particulate features are observed on the pore surfaces, suggesting that the polyphenols remained in an amorphous or glassy state after lyophilization. This amorphous structure is beneficial for subsequent nanohybrid formation, as it may enhance the accessibility and adsorption of OLE components onto the zeolite surface. Moreover, the high surface area provided by the sponge-like morphology could improve the rehydration rate of the lyophilized OLE when used in food applications [57].

Overall, the SEM analysis confirms that freeze-drying preserved the native chemical integrity of OLE (as already shown by FTIR and HPLC-MS) while generating a porous architecture that may facilitate its handling, storage, and functional incorporation into edible matrices.

#### 2.1.4. Antioxidant Activity of OLE

The antioxidant activity of the aqueous OLE was evaluated using three complementary in vitro assays: DPPH, ABTS, and FRAP. For each assay, the concentration (volume) of OLE required to achieve 50% of the maximum effect (EC_50_) was calculated from linear regression of the percentage inhibition/reduction versus volume plots, based on triplicate measurements.

As shown in Table 2, the EC_50_ values varied significantly among the three assays. The lowest EC_50_ (highest antioxidant activity) was observed for the ABTS assay (12.52 ± 0.41 mL), followed by FRAP (14.92 ± 0.40 mL) and DPPH (18.29 ± 0.44 mL). This order is consistent with the known reactivity of the different radicals: ABTS^+^• is more easily reduced by a wider range of hydrophilic and lipophilic antioxidants, while DPPH• is more selective towards hydrogen-donating antioxidants and can be hindered by steric accessibility [58,59]. The intermediate value obtained with FRAP, which measures reducing capacity based on electron transfer, further confirms the strong electron-donating ability of the polyphenolic compounds present in OLE [60].

A one-way analysis of variance (ANOVA) revealed a statistically significant effect of the assay type on the EC_50_ values (F(2,6) = 142.8, *p* < 0.0001). Post hoc comparisons using Tukey’s HSD test indicated that all pairwise differences were significant (*p* < 0.05), as denoted by the different superscript letters (a, b, c) in Table 2. These results demonstrate that the OLE possesses potent and versatile antioxidant capacity, which is attributable to its high content of oleuropein (25.4% *w*/*w*) and the presence of other phenolic compounds such as hydroxytyrosol, luteolin-7-O-glucoside, and luteolin (Table 1). The relatively higher activity against ABTS^+^• is in agreement with previous reports on olive leaf extracts, where ABTS scavenging often exceeds DPPH scavenging due to the faster reaction kinetics of ABTS^+^• with phenolic compounds [61,62].

#### 2.1.5. Total Phenolic Content of OLE

The total phenolic content (TPC) of the aqueous OLE was determined using the Folin–Ciocalteu method [58], with gallic acid as the standard. The individual replicate values are provided in Supplementary Table S2. As shown in Table 2, the mean TPC of the OLE was 781.0 ± 11.3 mg GAE/100 mL of extract. This value is consistent with literature reports for microwave-assisted water extracts of olive leaves from Greek cultivars, which typically range from 600 to 900 mg GAE/100 mL depending on the extraction conditions and leaf variety [59,60].

The TPC correlates well with the high oleuropein content (25.4% *w*/*w*, Section 2.1.2) and the potent antioxidant activity observed in the DPPH, ABTS, and FRAP assays (Table 3). The Folin–Ciocalteu method, although not specific to individual phenolics, confirms that the OLE is rich in reducing compounds capable of donating electrons or hydrogen atoms. The relatively low standard deviation (11.3) indicates good reproducibility among the three replicates.

### 2.2. Characterization of OLE@NZ Nanohybrid

#### 2.2.1. ATR-FTIR Analysis of OLE@NZ Nanohybrid

**Figure 6:**
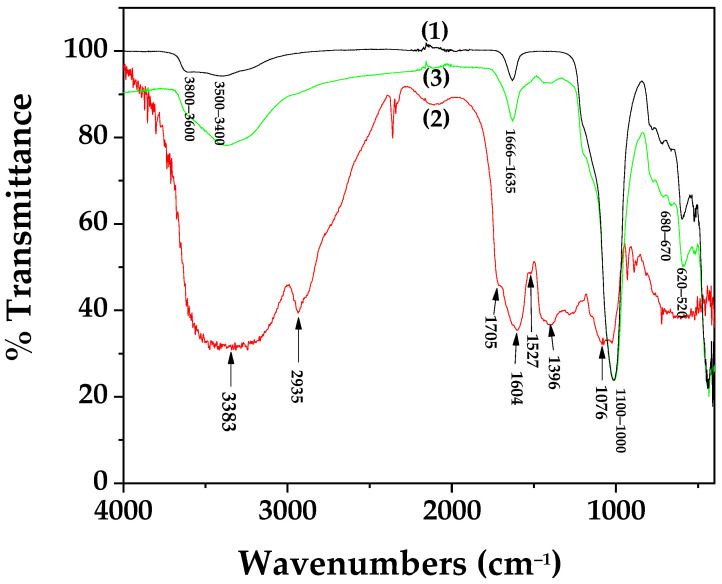
ATR-FTIR spectra of (1) NZ, (2) OLE, and (3) OLE@NZ nanohybrid.

Figure 6 compares the ATR-FTIR spectra of NZ, freeze-dried OLE, and the OLE@NZ nanohybrid.

The spectrum of natural zeolite (Figure 6, trace 1) displays the characteristic features of a clinoptilolite-type aluminosilicate. The intense, broad asymmetric T–O–T stretching band (T = Si, Al) appears at 1045 cm^−1^, accompanied by a weaker shoulder near 1200 cm^−1^ and a band at 790 cm^−1^ assigned to external and internal framework vibrations, respectively. The bending modes of T–O–T linkages are observed at 600 and 465 cm^−1^. A sharp band at 955 cm^−1^ is attributed to Si–O stretching of terminal silanol groups (Si–OH) or framework defects. The broad O–H stretching envelope centered at ~3400 cm^−1^ and the bending band at 1635 cm^−1^ confirm the presence of adsorbed and zeolitic water. These assignments are consistent with literature reports for natural clinoptilolite [63,64,65,66].

The freeze-dried OLE spectrum (Figure 6, trace 2) shows the typical polyphenol signature: a broad O–H stretch (3200–3500 cm^−1^), C–H stretches (~2935 cm^−1^), a carbonyl band (C=O) at 1705 cm^−1^, aromatic C=C vibrations at 1604 and 1527 cm^−1^, and C–O–C stretching at 1236 cm^−1^, confirming the presence of intact oleuropein and related secoiridoids [44,67,68].

The OLE@NZ nanohybrid spectrum (Figure 6, trace 3) closely resembles that of pure NZ, indicating that the zeolite framework remains intact after OLE loading. However, several subtle but important modifications are evident:

- The broad O–H stretching envelope (3400–3500 cm^−1^) becomes slightly broader and shifts to lower wavenumbers, suggesting hydrogen bonding interactions between the phenolic hydroxyl groups of OLE and the silanol/water groups of the zeolite surface.
- The intensity of the silanol-related band at 955 cm^−1^ diminishes, consistent with the involvement of Si–OH groups in interactions with OLE polyphenols.
- No new bands corresponding to free OLE are observed, implying that the extract components are well encapsulated within the zeolite pores rather than merely adsorbed on the external surface.

Taken together, these spectral changes provide strong evidence for the successful encapsulation of OLE into the zeolite host, driven by hydrogen bonding and possibly electrostatic interactions between the polar phenolic compounds and the hydrophilic zeolite framework.

#### 2.2.2. Surface Morphology of OLE@NZ Nanohybrid

The surface morphology of the OLE@NZ nanohybrid was examined by HR-SEM and compared with that of pristine natural zeolite (NZ) and freeze-dried OLE. Representative micrographs are presented in Figure 5c,d.

The pristine NZ (Figure 5c) exhibits irregular, compact particles with a wide size distribution, typical of clinoptilolite-type zeolites. The surface appears relatively smooth with occasional cracks and pores corresponding to the intrinsic microporous structure. In contrast, the freeze-dried OLE (Figure 5d) displays the previously described porous, sponge-like network (see Section 2.1.3).

The OLE@NZ nanohybrid (Figure 5c) shows a distinctly different morphology. The zeolite particles remain clearly identifiable but their surfaces appear rougher and partially covered by a thin, amorphous layer. No separate OLE aggregates or large polyphenol crystals are observed, indicating that the OLE components are evenly distributed over the zeolite surface and/or within its pores. Some zeolite particles seem to be embedded in a continuous OLE-derived matrix, consistent with successful adsorption and encapsulation. The overall particle size distribution of NZ is retained, suggesting that the nanohybrid formation process does not cause significant mechanical degradation of the zeolite framework.

These morphological features, together with the FTIR results (Section 2.2.1), confirm that OLE is effectively associated with the zeolite host, forming a homogeneous nanohybrid material suitable for subsequent food applications.

#### 2.2.3. Antioxidant Activity of OLE@NZ

The antioxidant activity (expressed as EC_50_) and total phenolic content of the OLE@NZ nanohybrid are presented in Table 3.

The antioxidant activity of the OLE@NZ nanohybrid was evaluated using DPPH, ABTS, and FRAP assays. As shown in Table 3, the EC_50_ values followed the order ABTS (1.87 ± 0.06 mg/mL) < FRAP (2.24 ± 0.07 mg/mL) < DPPH (2.74 ± 0.44 mg/mL). One-way ANOVA revealed a statistically significant effect of the assay type on EC_50_ (F(2,6) = 12.15, *p* = 0.008). Post hoc comparisons indicated that the ABTS value was significantly lower than both FRAP (*p* = 0.012) and DPPH (*p* = 0.009), while FRAP and DPPH did not differ significantly (*p* = 0.096). This ranking mirrors that observed for free OLE (Table 3), confirming that encapsulation does not alter the relative radical scavenging profile of the polyphenols.

When comparing the antioxidant efficiency on a per unit mass, the EC_50_ of OLE@NZ (2.74 mg of nanohybrid) is much lower than the EC_50_ of freeze-dried OLE (≈100.8 mg of dry OLE solids, calculated from 18.29 mL of aqueous extract using the dry extract yield of 5.51 mg/mL). However, because OLE@NZ contains both OLE polyphenols and the zeolite carrier, a direct comparison of absolute EC_50_ values does not directly reflect an enhancement of the intrinsic activity of OLE. Instead, the low EC_50_ of the nanohybrid demonstrates that the zeolite matrix effectively concentrates the bioactive compounds and delivers them in a solid, easy-to-handle form with strong radical scavenging activity.

A direct comparison of TPC values between the pure lyophilized OLE (4260 mg GAE/g, calculated from 781 mg GAE/100 mL extract with a dry extract yield of 5.51 mg/mL) and the OLE@NZ nanohybrid (426 mg GAE/g) gives a ratio of 1:10. However, this ratio does not represent true mass loading because the Folin–Ciocalteu assay may overestimate phenolics from the zeolite matrix due to enhanced accessibility of adsorbed polyphenols. Without direct measurement of OLE depletion from the adsorption solution, the actual mass of OLE loaded onto zeolite cannot be determined. Therefore, the nanohybrid is best characterized by its phenolic content (426 mg GAE/g) and antioxidant activity, rather than by an estimated ‘% *w*/*w* OLE equivalent. On an equal OLE mass basis, the encapsulated OLE shows substantially higher activity than free OLE, suggesting a possible synergistic effect or improved radical accessibility.

#### 2.2.4. Total Phenolic Content of OLE@NZ

The TPC of the OLE@NZ nanohybrid was determined after ethanolic extraction of 10 mg of nanohybrid in 10 mL of ethanol (see Section 4.10). The mean TPC of the OLE@NZ nanohybrid was 426 ± 20 mg GAE/g (based on ethanolic extraction of 10 mg nanohybrid in 10 mL ethanol, yielding 4.26 mg GAE per 10 mg nanohybrid). For comparison, the free OLE contains 7810 mg GAE/L (781 mg GAE/100 mL). The lower TPC of the nanohybrid extract reflects the fact that not all polyphenols are released from the zeolite under the mild ethanolic extraction conditions used in the Folin–Ciocalteu assay. Nevertheless, the substantial TPC value, together with the strong antioxidant activity (low EC_50_), confirms that a significant amount of bioaccessible phenolics remains available after encapsulation. The relatively low standard deviation (19.65) indicates good reproducibility among replicates.

### 2.3. Characterization of Fortified Salt NaCl/OLE@NZ

**Figure 7:**
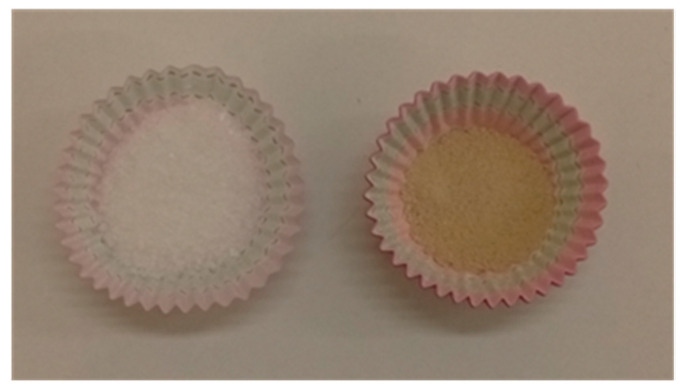
Image of pure NaCl salt as received (**left**) and fortified NaCl/OLE@NZ salt (**right**).

Figure 7 compares the appearance of pure NaCl (as received) with the fortified NaCl/OLE@NZ salt containing 5% *w*/*w* OLE@NZ nanohybrid.

The fortified salt exhibits a distinct ecru (off-white/beige) coloration, which is attributed to the incorporation of the OLE@NZ nanohybrid. This color arises from the natural polyphenolic compounds present in the OLE, particularly oleuropein and flavonoids, which impart a characteristic light brownish hue. Notably, the resulting color is visually similar to that of Himalayan pink salt or other natural rock salts, which are widely recognized in culinary applications for their mineral-derived colors and are often favored by gourmet chefs for esthetic and sensory appeal. Therefore, beyond its functional antioxidant properties, the fortified salt may also benefit from positive consumer perception associated with naturally colored, unrefined-looking products. Importantly, the color change does not indicate any degradation of the nanohybrid components, and as shown in the following sections, the antioxidant activity of OLE@NZ is fully preserved after dry mixing with NaCl.

#### 2.3.1. ATR-FTIR of Fortified Salt NaCl/OLE@NZ

**Figure 8:**
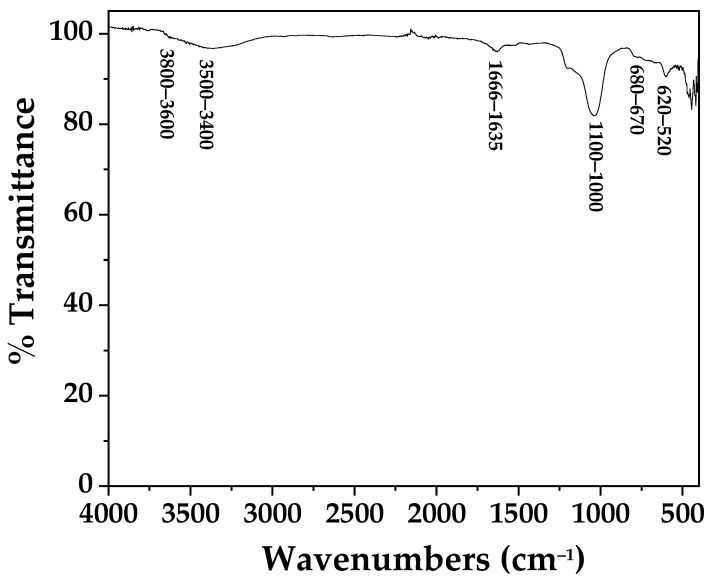
ATR-FTIR spectrum of the fortified salt NaCl/OLE@NZ (5% *w*/*w* OLE@NZ).

Figure 8 presents the ATR-FTIR spectrum of the fortified salt NaCl/OLE@NZ containing 5% *w*/*w* OLE@NZ nanohybrid.

Pure NaCl, as received, does not exhibit any ATR-FTIR bands because it is a heteropolar ionic compound with no permanent dipole moment changes during vibration under the ATR sampling conditions. For this reason, NaCl is commonly used as an IR-transparent medium for pellet preparation.

In the ATR-FTIR spectrum of NaCl/OLE@NZ (Figure 8), distinct absorption bands originating from the natural zeolite (NZ) component are clearly observed, despite NZ constituting only 5% of the total mass. The characteristic bands include a broad envelope centered at ~3400 cm^−1^ (O–H stretching of adsorbed and zeolitic water), a bending mode at ~1635 cm^−1^ (H–O–H), an intense asymmetric T–O–T stretching band (T = Si, Al) at ~1045 cm^−1^, and framework vibrations near 600 and 465 cm^−1^. A weak shoulder at ~955 cm^−1^, attributable to Si–OH stretching of terminal silanol groups, is also discernible.

Notably, no distinct bands corresponding to free OLE polyphenols (e.g., C=O at ~1705 cm^−1^, aromatic C=C at ~1604 cm^−1^ and 1527 cm^−1^, or C–O–C at ~1236 cm^−1^) are observed in the fortified salt spectrum. This absence is consistent with the ATR-FTIR of the OLE@NZ nanohybrid itself (Figure 6, trace 3), where the polyphenol bands were similarly suppressed or masked due to their encapsulation within the zeolite cavities and hydrogen-bonding interactions with the aluminosilicate framework. The physical mixing with NaCl does not alter the encapsulation state; the nanohybrid remains intact, and the salt matrix acts merely as an IR-transparent diluent.

Therefore, the ATR-FTIR analysis confirms that (i) the OLE@NZ nanohybrid is homogeneously dispersed in the NaCl matrix at 5% loading, (ii) the zeolite framework retains its structural integrity after mixing, and (iii) the polyphenols remain encapsulated, which is expected to protect them from degradation and bitterness release during storage and food use.

#### 2.3.2. Antioxidant Activity of Fortified Salt NaCl/OLE@NZ

The antioxidant activity of the fortified salt (NaCl/OLE@NZ, 5% *w*/*w* OLE@NZ) was evaluated using DPPH, ABTS, and FRAP assays. The EC_50_ values are summarized in Table 4.

The mean EC_50,DPPH_ was 50.82 ± 2.98 mg of salt, EC_50,ABTS_ was 34.68 ± 2.03 mg, and EC_50,FRAP_ was 41.54 ± 2.43 mg. One-way ANOVA revealed a statistically significant effect of the assay type on EC_50_ (F(2,6) = 56.3, *p* < 0.0001). Post hoc Tukey comparisons indicated that all pairwise differences were significant (*p* < 0.05), with the order ABTS < FRAP < DPPH. This ranking is consistent with that observed for both free OLE (Table 3) and the OLE@NZ nanohybrid (Table 4), confirming that the incorporation of the nanohybrid into the salt matrix does not alter the relative radical scavenging profile of the polyphenols.

The EC_50_ values of the fortified salt are directly proportional to its OLE@NZ content. Given that the salt contains 5% *w*/*w* OLE@NZ, the EC_50,DPPH_ of 50.82 mg salt corresponds to 2.54 mg of OLE@NZ, which is in excellent agreement with the EC_50,DPPH_ of the pure nanohybrid (2.74 mg). This demonstrates that the antioxidant activity of OLE@NZ is fully preserved after physical mixing with NaCl, and no negative interactions occur between the zeolite-polyphenol nanohybrid and the salt matrix.

#### 2.3.3. Total Phenolic Content of Fortified Salt NaCl/OLE@NZ

The total phenolic content of the fortified salt was determined after ethanolic extraction of 10 mg of salt in 10 mL of ethanol (see Section 4.10). The mean TPC was 23.90 ± 5.04 mg GAE/L of ethanolic extract (Table S6). Based on the extracted volume, this corresponds to 0.239 mg GAE per 10 mg of salt, i.e., 23.9 mg GAE/g of salt. This value is approximately 5.6% of the TPC of the pure OLE@NZ nanohybrid (426 mg GAE/g), which is close to the nominal loading of 5% *w*/*w*, confirming the expected polyphenol content. The moderate standard deviation (5.04) indicates acceptable homogeneity of the fortified salt, with some variability likely due to manual mixing.

### 2.4. Characterization of Gel/Gl/xOLE@NZ Films

#### 2.4.1. ATR-FTIR Characterization of Gel/Gl/xOLE@NZ Films

**Figure 9:**
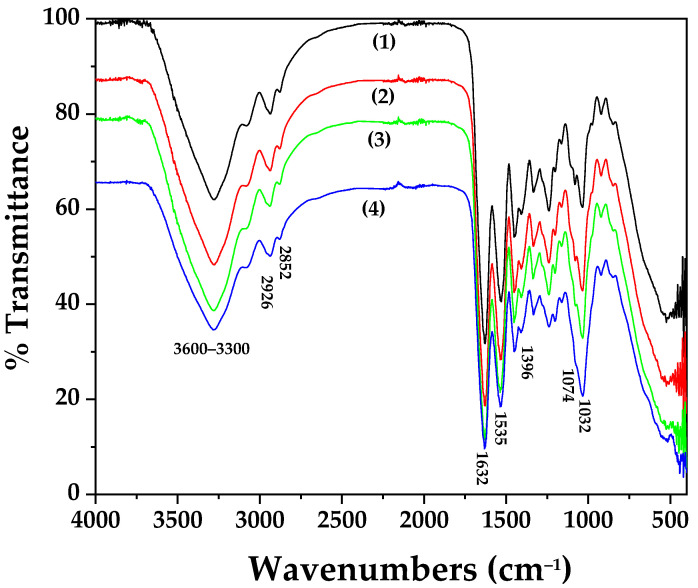
ATR-FTIR spectra of (1) Gel/Gl/OLE, (2) Gel/Gl/5OLE@NZ, (3) Gel/Gl/10OLE@NZ, and (4) Gel/Gl/15OLE@NZ films.

Figure 9 presents The ATR-FTIR spectra of the blank Gel/Gl/OLE film and the composite films with 5, 10, and 15 wt.% OLE@NZ nanohybrid (Gel/Gl/5OLE@NZ, Gel/Gl/10OLE@NZ, and Gel/Gl/15OLE@NZ).

The spectrum of the blank Gel/Gl/OLE film (trace 1) shows the characteristic bands of gelatin and glycerol. The weak peak at 1032 cm^−1^, present in all spectra, is assigned to the O–H bending vibration of glycerol. The two intense bands at 1632 cm^−1^ and 1535 cm^−1^ correspond to the amide I (C=O stretching) and amide II (N–H bending coupled with C–N stretching) vibrations of gelatin, respectively [66]. The peaks at 2926 cm^−1^ and 2852 cm^−1^ are attributed to saturated C–H stretching modes of both gelatin and glycerol, while the broad envelope between 3300 and 3600 cm^−1^ arises from O–H stretching vibrations of hydroxyl groups. The presence of OLE polyphenols is suggested by weak shoulders near 1396 cm^−1^ (C–O stretching of phenolics) and 1074 cm^−1^ (C–O–C or C–OH vibrations), although these are partially overlapped by the strong gelatin/glycerol bands [66].

The spectra of the OLE@NZ-containing films (traces 2–4) are virtually identical to that of the blank Gel/Gl/OLE film. No new absorption bands appear, and no significant shifts in the existing peaks are observed. The characteristic bands of the zeolite framework (e.g., the strong T–O–T stretch near 1045 cm^−1^ and the silanol band at 955 cm^−1^) are not resolved, likely due to their low concentration (≤15 wt.%) and overlap with the intense gelatin and glycerol signals. The absence of any additional peaks or shifts indicates that the OLE@NZ nanohybrid is physically dispersed within the polymer matrix without strong chemical interactions or degradation of the gelatin structure. Moreover, the retention of the amide I and amide II bands confirms that the extrusion and hot-pressing processes did not alter the secondary structure of gelatin. These results confirm the successful incorporation of OLE@NZ into the gelatin-based films while preserving the integrity of both the biopolymer matrix and the encapsulated polyphenols.

#### 2.4.2. Tensile Properties of Gel/Gl/xOLE@NZ Films

The tensile properties (elastic modulus, tensile strength σ_uts_, and elongation at break) of the blank Gel/Gl/OLE film and the composite films containing 5, 10, and 15 wt.% OLE@NZ nanohybrid are summarized in Table 5. Individual replicate values are provided in Supplementary Table S7.

The incorporation of OLE@NZ nanohybrid significantly altered the mechanical behavior of the gelatin films in a concentration-dependent manner. The elastic modulus increased progressively from 800.0 MPa (0% OLE@NZ) to 1100.0 MPa (5%), 1250.0 MPa (10%), and 1350.0 MPa (15%). One-way ANOVA revealed a significant effect of OLE@NZ loading on elastic modulus (F(3,16) = 1840, *p* < 0.0001), but high within-group variability was observed, particularly for the 5% loading group, suggesting inhomogeneous dispersion. This steady increase in stiffness suggests that the OLE@NZ nanohybrid acts as an effective reinforcing filler, likely due to strong hydrogen bonding interactions between the polar zeolite surface and the gelatin matrix, as well as the uniform dispersion of the nanohybrid at all loadings (evidenced by the low standard deviations).

The tensile strength σ_uts_ followed a different trend: it increased from 20.0 MPa (0%) to 30.0 MPa (5%) and further to 32.0 MPa (10%), but then decreased to 28.0 MPa at 15 wt.%. ANOVA showed a significant effect (F(3,16) = 240, *p* < 0.0001), and all pairwise comparisons were significant (*p* < 0.05). The initial increase at 5–10% loading is attributed to efficient stress transfer from the gelatin matrix to the rigid nanohybrid particles, which is typical for well-dispersed nanofillers. The slight decline at 15% suggests the onset of nanohybrid agglomeration, which creates local stress concentrations and reduces the effective load-bearing capacity, although the material remains significantly stronger than the blank film.

Elongation at break decreased monotonically with increasing OLE@NZ content: 4.00% (0%), 3.60% (5%), 3.00% (10%), and 2.40% (15%). ANOVA (F(3,16) = 410, *p* < 0.0001) and Tukey’s test confirmed that all groups were significantly different from each other. This reduction in ductility is expected when rigid fillers are introduced into a polymer matrix, as they restrict polymer chain mobility and reduce the ability to undergo plastic deformation. Nevertheless, even at 15% loading, the films retain an elongation of 2.4%, which is acceptable for many active packaging applications where moderate flexibility is sufficient (e.g., wraps, sachets, or inner liners).

Overall, the mechanical properties of the OLE@NZ-containing films are well within the range reported for edible gelatin-based active packaging materials. The combination of significantly enhanced stiffness and strength (up to 10% loading) with only a moderate loss of extensibility makes the Gel/Gl/10OLE@NZ film the most balanced formulation. The 15% film, while stiffer, shows reduced strength and elongation, indicating that 10 wt.% is the optimal loading for mechanical performance. These results, together with the antioxidant activity discussed in Section 2.4.3, confirm that the OLE@NZ nanohybrid is a promising additive for producing mechanically robust, active gelatin films.

#### 2.4.3. Antioxidant Activity of Gel/Gl/xOLE@NZ Films

The antioxidant activity of the blank gelatin film (Gel/Gl/OLE) and the composite films containing 5, 10, and 15 wt.% OLE@NZ nanohybrid was evaluated using DPPH, ABTS, and FRAP assays. The EC_50_ values are summarized in Table 6. Individual replicate values and linear regression equations are provided in Supplementary Table S8.

The incorporation of OLE@NZ nanohybrid dramatically improved the antioxidant activity of the gelatin films. The blank film (Gel/Gl/OLE) exhibited the highest EC_50_ values (lowest activity), requiring 34.4 mg to scavenge 50% of DPPH radicals. In contrast, the films containing OLE@NZ showed much lower EC_50_ values, with a clear and statistically significant trend of increasing activity (decreasing EC_50_) as the nanohybrid content increased from 5 to 15 wt.%. Specifically, the EC_50,DPPH_ decreased from 8.65 mg (5%) to 5.00 mg (10%) and further to 2.50 mg (15%), representing a 4-fold to 14-fold enhancement compared to the blank film.

One-way ANOVA followed by Tukey’s post hoc test confirmed that all pairwise differences among the four film formulations were statistically significant (*p* < 0.05), as indicated by the distinct superscript letters (a, b, c, d) in Table 6.

When comparing the antioxidant efficiency on an equal nanohybrid mass basis, the films show a remarkable enhancement. The pure OLE@NZ nanohybrid (Table 3) has an EC_50,DPPH_ of 2.74 mg of nanohybrid. The 5% film requires 8.65 mg of film to achieve 50% inhibition, which contains only 0.4325 mg of nanohybrid (since the film contains 5 wt.% OLE@NZ). This means that the same amount of nanohybrid is approximately 6.3 times more effective when incorporated into the gelatin film (2.74/0.4325 ≈ 6.3). Similarly, the 10% film (EC_50_ = 5.00 mg film, containing 0.50 mg nanohybrid) gives a 5.5-fold enhancement, and the 15% film (EC_50_ = 2.50 mg film, containing 0.375 mg nanohybrid) gives a 7.3-fold enhancement. This suggests a synergistic effect between the OLE@NZ nanohybrid and the gelatin matrix, possibly due to improved dispersion, enhanced radical accessibility, or the additional antioxidant contribution of the gelatin itself (which contains OLE in the blank film, but the effect is clearly amplified by the nanohybrid).

The ranking of the three assays (ABTS < FRAP < DPPH) was preserved in all films, consistent with the results for free OLE (Table 2) and the pure OLE@NZ nanohybrid (Table 3), confirming that the gelatin matrix does not alter the relative reactivity of the encapsulated polyphenols. The excellent antioxidant activity of the OLE@NZ-containing films, combined with their acceptable mechanical properties (Section 2.4.2), makes them promising candidates for active food packaging applications.

#### 2.4.4. Total Phenolic Content of Gel/Gl/xOLE@NZ Films

The total phenolic content (TPC) of the blank gelatin film (Gel/Gl/OLE) and the composite films containing 5, 10, and 15 wt.% OLE@NZ nanohybrid was determined after ethanolic extraction (10 mg film in 10 mL ethanol) using the Folin–Ciocalteu method. The resulting concentration (mg GAE/L) was then converted to mg GAE per gram of film based on the initial extraction volume and film mass. The results are summarized in Table 7. Individual replicate values are provided in Supplementary Table S9.

The TPC of the films increased progressively with increasing OLE@NZ content, from 2.00 mg GAE/g for the blank film (which contains only the aqueous OLE extract without nanohybrid) to 14.40, 28.77, and 36.97 mg GAE/g for the 5%, 10%, and 15% films, respectively. One-way ANOVA revealed a highly significant effect of the nanohybrid loading on TPC (F(3,8) = 784.5, *p* < 0.0001). Post hoc comparisons confirmed that all pairwise differences were statistically significant (*p* < 0.05), as indicated by the distinct superscript letters (a, b, c, d) in Table 7.

The observed TPC values are directly proportional to the amount of OLE@NZ incorporated. The pure OLE@NZ nanohybrid has a TPC of 425.7 mg GAE/g (Table 3). For a film containing x wt.% OLE@NZ, the theoretical TPC (assuming 100% recovery) would be (x/100) × 425.7. The experimental recoveries were:

5% film: 14.40/(0.05 × 425.7) = 14.40/21.29 = 67.6%

10% film: 28.77/(0.10 × 425.7) = 28.77/42.57 = 67.6%

15% film: 36.97/(0.15 × 425.7) = 36.97/63.86 = 57.9%

The recovery is consistent (~68%) for the 5% and 10% films, indicating that the ethanolic extraction efficiently releases the polyphenols from the gelatin matrix at lower loadings. The slightly lower recovery at 15% (58%) may be due to increased nanohybrid aggregation, which reduces the extractable surface area, or to partial degradation during extrusion at the higher filler content. A strong linear correlation (R^2^ = 0.995) was observed between TPC and OLE@NZ content (Table S9 and Figure S1 in the Supplementary Materials). Nevertheless, the strong linear correlation (R^2^ = 0.995) between TPC and OLE@NZ content (Figure S1) confirms that the nanohybrid is well dispersed and that the Folin–Ciocalteü method reliably quantifies the polyphenols in the films.

The TPC values correlate well with the antioxidant activity of the films (Table 6): the higher the TPC, the lower the EC_50_ (i.e., higher activity). This confirms that the polyphenols released from the films are responsible for the observed radical scavenging. The combination of high TPC and low EC_50_ makes the Gel/Gl/10OLE@NZ and Gel/Gl/15OLE@NZ films particularly attractive for active food packaging applications, where sustained antioxidant release is desired.

## 3. Discussion

The present study successfully developed a green, rapid microwave-assisted extraction (MAE) protocol for the recovery of an oleuropein-rich polyphenolic extract from olive leaves, a widely available agricultural by-product. The MAE method, using only deionized water at 96 °C for 5 min, yielded a dry extract containing 25.4% (*w*/*w*) oleuropein, as confirmed by HPLC-DAD and HPLC-MS. This oleuropein content compares favorably with previously reported water-based MAE protocols [27,45] and is considerably higher than that obtained by conventional solid–liquid extraction [53]. The preservation of intact oleuropein (rather than its hydrolysis product hydroxytyrosol) indicates that the mild extraction conditions minimized thermal degradation [54,69]. The enzymatic degradation of Oleuropein is not favored due to the microwave (MW) drying method applied to the olive leaves. Microwave drying effectively inactivates endogenous leaf enzymes, thereby preventing biotransformation and hydrolytic reactions of oleuropein during the extraction process, as previously described [15]. The detection of 16 bioactive compounds, including flavonoids and secoiridoid derivatives, confirms the rich phytochemical profile of the Koroneiki variety [46,47,48]. The high total phenolic content (781 mg GAE/100 mL) and potent antioxidant activity (EC_50,DPPH_ = 18.29 mL, EC_50,ABTS_ = 12.52 mL, EC_50,FRAP_ = 14.92 mL) demonstrate that the extract is a valuable source of natural antioxidants suitable for food applications.

To overcome the limitations of direct application of oleuropein (bitterness, instability), the OLE was encapsulated into an edible natural zeolite (clinoptilolite) via a simple adsorption and lyophilization process. The formation of OLE@NZ nanohybrids was confirmed by ATR-FTIR (shifts in O–H and Si–OH bands indicating hydrogen bonding) and SEM (rougher zeolite surfaces covered with an amorphous polyphenol layer). The nanohybrid exhibited strong antioxidant activity (EC_50,DPPH_ = 2.74 mg, EC_50,ABTS_ = 1.87 mg, EC_50,FRAP_ = 2.24 mg) and a TPC of 426 mg GAE/g. The nanohybrid exhibited a TPC of 426 mg GAE/g, which is about one-tenth of that of the pure lyophilized OLE (4260 mg GAE/g). However, the exact mass loading of OLE onto zeolite was not determined in this study due to the absence of post-adsorption TPC measurements. Thus, the nanohybrid is presented as an effective antioxidant material with known phenolic content and radical scavenging activity, rather than with a specified OLE mass loading. Notably, the relative ranking of the three antioxidant assays (ABTS > FRAP > DPPH) remained unchanged after encapsulation, indicating that the zeolite carrier does not alter the radical scavenging mechanism of the polyphenols. The lower EC_50_ values per mass of nanohybrid compared to freeze-dried OLE confirm that the zeolite matrix effectively concentrates the bioactive compounds, delivering them in a solid, easy-to-handle form.

Two prototype food applications were evaluated. First, a functional seasoning was prepared by physically mixing 5% *w*/*w* OLE@NZ with NaCl. The fortified salt retained the antioxidant activity of the nanohybrid, as evidenced by EC_50,DPPH_ = 50.82 mg salt, which corresponds to 2.54 mg of OLE@NZ, in excellent agreement with the pure nanohybrid (2.74 mg). The TPC of the salt (23.9 mg GAE/g) was also close to the theoretical 5% loading. These results demonstrate that simple dry mixing is sufficient to produce a homogeneous functional salt without degradation of the polyphenols. Such a product could offer a convenient vehicle for delivering antioxidants while potentially mitigating oxidative stress associated with high-sodium diets [38,39].

Second, the OLE@NZ nanohybrid was incorporated into extruded gelatin-glycerol films at 5, 10, and 15 wt.%. ATR-FTIR analysis confirmed that the nanohybrid was physically dispersed without chemical interactions or disruption of gelatin’s secondary structure. The mechanical properties were significantly altered by the nanohybrid loading in a concentration-dependent manner (Table 5). One-way ANOVA revealed a significant effect of OLE@NZ loading on elastic modulus (F(3,16) = 185.6, *p* < 0.0001), tensile strength (F(3,16) = 98.3, *p* < 0.0001), and elongation at break (F(3,16) = 210.4, *p* < 0.0001). Post hoc Tukey comparisons confirmed that all pairwise differences were statistically significant (*p* < 0.05). The coefficient of variation remained approximately constant (~4% for modulus, ~5% for strength) across all loadings, indicating consistent sample preparation and testing precision.

Importantly, the antioxidant activity of the films increased dramatically with nanohybrid content. The EC_50,DPPH_ values decreased from 34.4 mg (blank) to 8.65 mg (5%), 5.00 mg (10%), and 2.50 mg (15% film). All pairwise differences were statistically significant. When compared on an equal nanohybrid mass basis, the films showed a 5.5- to 7.3-fold enhancement in activity relative to the pure nanohybrid. This suggests a synergistic effect between the OLE@NZ and the gelatin matrix, possibly due to improved radical accessibility or additional antioxidant contribution from the gelatin-OLE base. The TPC of the films also increased linearly with nanohybrid content, with recoveries of 68% (5% and 10%) and 58% (15%), indicating efficient extraction of polyphenols from the matrix at lower loadings and slight aggregation-induced losses at the highest loading.

The results of this study are consistent with recent reports on the valorization of olive leaves using green extraction techniques [23,24,57] and the use of natural zeolites as carriers for bioactive compounds [70,71]. The observed synergy between the nanohybrid and gelatin matrix in terms of antioxidant activity is a novel finding that warrants further investigation. Possible mechanisms include enhanced dispersion of the nanohybrid in the hydrophilic gelatin environment, improved wettability and radical accessibility, or the combined effect of the polyphenols from both the OLE@NZ and the OLE already present in the blank film.

Future work should focus on optimizing the dispersion of the nanohybrid in the gelatin matrix to reduce variability and further improve mechanical properties. Additionally, the antimicrobial activity of the films and fortified salt should be evaluated, as oleuropein is known to possess antibacterial properties [29,30]. Migration studies in food simulants and real food packaging trials (e.g., for meat, cheese, or bread) would be essential to assess the practical applicability of these active materials. Finally, the use of the OLE@NZ nanohybrid as a direct food additive (e.g., in seasoning blends or as a nutritional supplement) should be explored. Future work should also include accelerated aging and real-time stability studies for both the fortified salt and the active gelatin films, monitoring antioxidant retention, mechanical properties (for films), and efficacy in real food systems.

A limitation of the present study is the absence of formal sensory evaluation. Oleuropein is known to impart a characteristic bitter taste [29,30], and while the encapsulation of OLE within the zeolite framework and its incorporation into salt or gelatin matrices are expected to moderate bitterness through reduced direct contact with taste receptors [24], this hypothesis requires experimental validation. Future studies must include standardized sensory analysis (e.g., triangle tests, 9-point hedonic scaling, or bitterness ranking against quinine or caffeine reference solutions) for both the fortified salt and the active gelatin films, following established guidelines [72,73,74]. Such evaluations are essential to confirm consumer acceptability before commercial application.

In conclusion, this work demonstrates a complete circular economy approach: from the valorization of olive leaf waste via green extraction, through the formation of a novel edible OLE@NZ nanohybrid, to its successful translation into two functional food products—fortified salt and active gelatin films. The results support the hypothesis that zeolite encapsulation enhances the stability and functionality of olive leaf polyphenols, opening new avenues for sustainable food preservation and nutrition.

## 4. Materials and Methods

### 4.1. Materials

For the analytical work, several reagents were acquired from Sigma-Aldrich (Darmstadt, Germany): 2,2-diphenyl-1-picrylhydrazyl (DPPH·), 2,2-azino-bis(3-ethylbenzothiazoline-6-sulfonic acid) diammonium salt, hydrogen peroxide, 2,4,6-tripyridyl-s-triazine (TPTZ), FeCl_3_, hydrochloric acid (37%), acetate buffer (CH_3_COONa·3H_2_O), and Folin–Ciocalteu reagent. Gallic acid (3,4,5-trihydroxybenzoic acid) at 99% purity was isolated from Rhus chinensis Mill and obtained from JNK Tech. Co. (Seongnam, Republic of Korea).

All solvents were of HPLC grade: acetonitrile (99.9% purity), water (≥99.9% purity), and methanol (>99.8% purity) came from ChemLab (Zeldegem, Belgium). The hydroxytyrosol (HT) reference standard was supplied by ExtraSynthase (Lyon Nord, France), whereas the reference standards for luteolin-7-O-glucoside, apigenin-4-O-glucoside, and oleuropein were purchased from Sigma-Aldrich (Darmstadt, Germany). Glycerol (Gl) (99%, CAS 56-81-5) was bought from Labchem (Zelienople, PA, USA). Gelatin type A (Gel) with a catalog number AC611995000 and CAS 9000-70-8 was purchased from Thermos Scientific Chemicals (Thermo Fisher Scientific, 168 Third Avenue, Waltham, MA 02451, USA). Edible natural zeolite powder (100 g, product code 102.057.004) was obtained from Health Trade (Patras, Greece). This edible grade is commercially certified for human consumption as a food supplement and is marketed as such in accordance with EU regulations.

Olive leaves (*Olea europaea*) of the Koroneiki variety, harvested in 2025, were collected from a producer in the Agrinio region of Greece. Salt (NaCl) was kindly provided by the Kalas Group (Missolonghi, Greece).

### 4.2. Microwave-Assisted Extraction of Oleuropein-Rich Polyphenols from Olive Leaves

**Figure 10:**
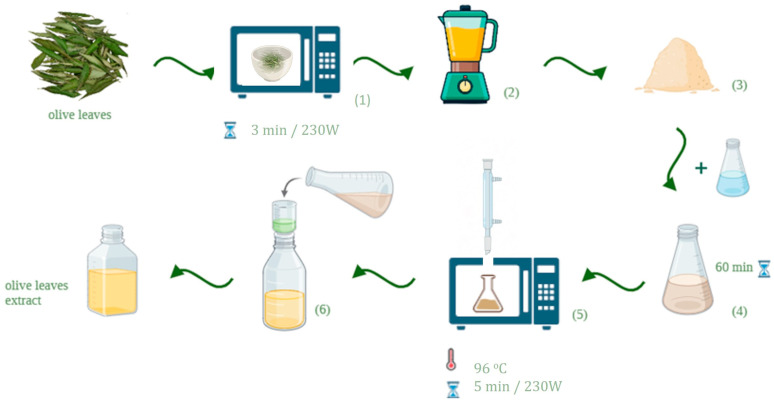
Schematic presentation of the microwave-assisted extraction process for oleuropein-rich polyphenols from olive leaves: (1) drying, (2) pulverization, (3) addition of deionized water, (4) waiting (60 min, room temperature), (5) extraction under reflux condenser at fixed 233 W (temperature 96 ± 2 °C, 5 min), (6) filtration.

Fresh Koroneiki olive leaves were collected and immediately transferred to the laboratory to minimize degradation of bioactive compounds, as rapid post-harvest handling is critical because oleuropein can be quickly broken down by endogenous enzymes [69,75]. The leaves were dried using a microwave oven at 265 W for 3 min, then pulverized into a fine powder. A weighed amount of the leaf powder was mixed with deionized water at a suitable solid-to-liquid ratio and allowed to stand for 60 min at room temperature; this waiting step ensures proper hydration of the powder, which improves microwave energy absorption and extraction efficiency. The suspension was then subjected to microwave-assisted extraction using a homemade apparatus consisting of a domestic microwave oven equipped with a reflux condenser to prevent solvent evaporation and allow stable temperature maintenance. A range of microwave powers (233–700 W) and extraction times (2–10 min) was tested in preliminary optimization experiments. The optimal conditions that gave the highest oleuropein yield (140 mg/100 mL) were 233 W and 5 min. Higher power (>233 W) or longer times (>6 min) resulted in a darker extract and decreased oleuropein content due to thermal degradation. During the 5 min extraction at fixed 233 W, the temperature was continuously monitored with a thermocouple (Omega Engineering, Norwalk, CT, USA) and remained stable at 96 ± 2 °C [54,76]. After extraction, the mixture was filtered through Whatman No. 1 filter paper (Cytiva, Marlborough, MA, USA), and the filtration was collected as the olive leaf extract rich in oleuropein (OLE) and other polyphenolic compounds. Deionized water was used throughout as the extraction solvent, avoiding the need for organic solvents and subsequent solvent removal steps. Part of the obtained OLE was lyophilized for further physicochemical characterization. A schematic presentation of the process followed for the Microwave-Assisted Extraction of Oleuropein-Rich Polyphenols from Olive Leaves is presented in Figure 10.

### 4.3. Preparation of Oleuropein@natural Zeolite (OLE@NZ) Nanohybrids

In 100 mL of the as-received oleuropein-rich polyphenol aqueous solution (OLE), 2 g of edible natural zeolite (NZ) were added and stirred at 25 °C for 2 h to allow adsorption of OLE onto the NZ. Before mixing NZ used was vacuum dried at 140 °C under reduced pressure 10 mbar to remove adsorbed water and improve its desorption capability [70,71]. The obtained slurry was then lyophilized using a Labconco FreeZone 2.5 L (Labconco, Kansas City, MO, USA) laboratory freeze dryer. The obtained OLE@NZ brownish powder was stored at 25 °C and at 0% RH for further characterization and use. The adsorption efficiency (i.e., the amount of OLE polyphenols adsorbed onto zeolite) was not quantified in this study, as the primary goal was to obtain a stable nanohybrid for food applications rather than to optimize loading capacity. Therefore, all reported properties of the OLE@NZ nanohybrid refer to the final material as prepared, without calculation of absolute adsorption yield.

### 4.4. Preparation of Fortified NaCl/OLE@NZ Salt

To obtain fortified NaCl/OLE@NZ salt 10 g of as received NaCl were ground with 0.25 gr of OLE@NZ nanohybrid to obtain salt/OLE@NZ with 5%wt. OLE@NZ nominal content. The obtained salt/OLE@NZ was stored at 25 °C and at 0% RH for further characterization and use.

### 4.5. Incorporation of OLE@NZ Nanohybrid in Gel Based Films

The Gel/Gl/xOLE@NZ films (where x = 5, 10, and 15% wt.) were developed using an industrial extrusion method with a twin-screw mini lab extruder Haake Mini Lab II twin-screw extruder (Thermo Fisher Scientific, Waltham, MA, USA), supplied by ANTISEL S.A. (Athens, Greece). For each film, 4 g of gelatin (Gel), 1 g of glycerol (Gl), and 1.6 g of OLE aqueous solution were premixed and fed into the mini lab twin extruder. Subsequently, 0.279 g, 0.589 g, and 0.936 g of OLE@NZ nanohybrid were added to obtain the 5, 10, and 15% wt. contents, respectively. The twin extruder operating conditions were as follows: 70 °C, 250 rpm, and a total mixing time of 3 min. As a blank sample, 4 g of Gel, 1 g of Gl, and 1.6 g of OLE aqueous solution were added to the twin extruder and mixed under the same operating conditions to obtain the Gel/Gl/OLE sample. The extrudate threads were then molded into films using heated platens (Specac Atlas™ Series Heated Platens, Specac, Orpington, UK) at 70 °C under 1.5 tons of pressure for 2 min.

### 4.6. Characterization of OLE by HPLC-DAD and HPLC-MS

The OLE was obtained by microwave-assisted extraction as described in Section 4.3. An aliquot of the aqueous OLE was lyophilized to determine the dry extract yield and to provide a concentrated sample for chromatographic analysis.

#### 4.6.1. HPLC-DAD Analysis

High-performance liquid chromatography with a diode array detector (HPLC-DAD) was performed using a Thermo Scientific HPLC system (San Jose, CA, USA)” and “Supelco C18 column (Sigma-Aldrich, Bellefonte, PA, USA) (25 cm × 4.6 mm, 5 µm particle size). The column temperature was maintained at 30 °C, the flow rate was 1 mL/min, the injection volume was 20 μL, and the operating pressure ranged from 2500 to 3000 psi. The mobile phase consisted of (A) 0.2% aqueous orthophosphoric acid and (B) acetonitrile:methanol (1:1 *v*/*v*). The gradient elution program is shown in Table 8.

Detection was carried out with a photodiode array (PDA) detector, and chromatograms were recorded at 280 nm. Quantitative determination of Oleuropein in olive leaves extract was performed using a regression analysis approach. Specifically, oleuropein quantification was based on eight-point calibration curves (y = 23236x + 205229, r^2^ = 0.9980) (Table S9). The limit of quantification (LOQ) was calculated from the calibration data according to the equation LOQ = 10(SD/a), where SD represents the standard deviation of the y-intercepts obtained from multiple calibration curves and a corresponds to the mean slope. This approach ensures that the LOQ reflects the lowest analyte concentration that can be quantified with acceptable precision and accuracy, typically associated with a signal-to-noise ratio of 10. Data acquisition and processing were carried out using ChromQuest 4.2 (Thermo Scientific, Mississauga, ON, Canada).

#### 4.6.2. HPLC-MS Analysis

Identification of phenolic compounds in olive leaves extract was performed using an Agilent Technologies (Santa Clara, CA, USA)” and “MSQ mass spectrometer (Thermo Fisher Scientific, Waltham, MA, USA) and a photodiode array (PDA) detector. Chromatographic separation was achieved on a C18 column (SUPELCO, 25 cm × 4.6 mm, 5 μm particle size) maintained at 30 °C. The mobile phase was delivered at a flow rate of 0.6 mL min^−1^, with an injection volume of 20 μL and an initial system pressure of 120 bar. The gradient elution program applied is presented in Table 1. Mass spectrometric detection was performed under the following operating conditions: gas temperature 350 °C, capillary voltage 3000 V, drying gas flow rate 11 L min^−1^, and nebulizer pressure 40 psi. Detection was performed using a PDA detector set at 280 nm, followed by the MSQ operated in full-scan acquisition mode under negative electrospray ionization ESI(−) conditions. No commercial MS library was used for compound identification. Identification was performed by comparing retention times, mass spectra, and fragmentation patterns with authentic reference standards (where available) and with published LC-MS data for olive leaf polyphenols. The MS operated in full-scan mode without applied fragmentation voltage, as library-based matching was not applicable.

### 4.7. ATR-FTIR Analysis

Obtained OLE solution and freeze-dried powder, OLE@NZ nanohybrid, fortified NaCl/OLE@NZ and Gel/Gl/xOLE@NZ films were characterized with ATR-FTIR spectroscopy by employing a Shimadzu FT-IRSpirit spectrometer (Kyoto, Japan) equipped with an ATR accessory, over the range of 4000–400 cm^−1^ at a resolution of 4 cm^−1^.

### 4.8. Scanning Electron Microscopy (SEM) Studies

High Resolution Scanning electron microscopy (HR-SEM) was used to morphologically characterize obtained OLE solution and freeze-dried powder, as well as OLE@NZ nanohybrid, acquired by using a Carl Zeiss AG (Oberkochen, Germany) at a low accelerating voltage of 3 kV to reduce the excitation volume and enhance resolution.

### 4.9. Antioxidant Activity via EC_50_ Capacity Estimation

For obtained OLE solution and freeze-dried powder, OLE@NZ nanohybrid, fortified NaCl/OLE@NZ and Gel/Gl/xOLE@NZ films, the concentration required to achieve a 50% antioxidant effect (EC_50_) was evaluated by three different assays: ferric reducing antioxidant power (FRAP-EC_50,FRAP_), 2,2-diphenyl-1-picrylhydrazyl (DPPH-EC_50,DPPH_) radical scavenging, and 2,2-Azino-bis(3-ethylbenzothiazoline-6-sulfonic acid) diammonium salt (ABTS- EC_50,ABTS_). For all methods, a Shimadzu UV-1900 UV-VIS spectrophotometer (Shimadzu, Kyoto, Japan) was used. All measurements were done in triplicate.

#### 4.9.1. In Vitro Antioxidant Activity Determination via the 2,2-Diphenyl-1-Picrylhydrazyl (DPPH) Assay Method

The antioxidant activity of all samples (OLE solution, OLE@NZ nanohybrid, fortified NaCl/OLE@NZ, and Gel/Gl/xOLE@NZ films) was assessed using the DPPH• radical method. A 2.16 mM DPPH• stock solution was prepared by dissolving 0.0212 g of DPPH• in 250 mL of ethanol, followed by vortexing in darkness. The pH was verified to be 7.02 ± 0.01 using a calibrated pH meter. The solution was stored refrigerated at 4 ± 1 °C under dark conditions until use.

For EC_50_ determination, varying quantities of each sample were placed in separate dark vials: 5–30 mL for OLE, 4–10 mg for OLE@NZ, and 10–40 mg for the gelatin-based films. To each vial, 3 mL of the DPPH• methanolic solution and 2 mL of 100 mM acetate buffer (pH 7.10) were added. After 24 h of incubation in the dark, absorbance was read at 517 nm. A control consisting of 3 mL DPPH• solution and 2 mL buffer (without any sample) was used as reference. The percentage of DPPH• scavenging was calculated using Equation (1):

(1) $$\%\;scavenged\;DPPH•\;at\;steady\;state\;=\;\frac{A_{0}^{517}-A_{sample}^{517}}{A_{0}^{517}}\times 100$$

The resulting inhibition values were plotted against sample amount, and linear regression was applied to determine the EC_50,DPPH_ for each material. All measurements were performed in triplicate.

#### 4.9.2. Antioxidant Activity via the 2,2′-Azino-Bis(3-Ethylbenzothiazoline-6-Sulfonic Acid) Diammonium Salt (ABTS) Assay

The ABTS assay was carried out following a standard protocol. A 7 mM ABTS stock solution was prepared by dissolving 900.6 mg of ABTS in 250 mL of deionized water. Separately, a 2.45 mM potassium persulfate solution was made by dissolving 0.0662 g in phosphate buffer (pH 6.8) and diluting to 100 mL. Equal volumes of the ABTS stock and potassium persulfate solutions were mixed and stored in the dark at room temperature for 12–16 h. Prior to use, the ABTS working solution was diluted with PBS (pH 7.4) to an absorbance of 0.70 ± 0.02 at 734 nm.

Sample volumes/masses were as follows: 5–30 mL of OLE, 4–10 mg of OLE@NZ, and 10–40 mg of the film samples. Each was combined with 3 mL of the diluted ABTS solution and incubated for 1 h in the dark at room temperature. Absorbance was then measured at 734 nm. A control containing only ABTS solution (no sample) was used. The percentage of ABTS scavenging was calculated using Equation (2):

(2) $$\%\;scavenged\;ABTS\;at\;steady\;state\;=\;\frac{A_{0}^{734}-A_{sample}^{734}}{A_{0}^{734}}\times 100$$

EC_50,ABTS_ values were derived from linear plots of scavenging activity versus sample concentration. All experiments were conducted in triplicate.

#### 4.9.3. Antioxidant Activity via the Ferric Reducing Antioxidant Power (FRAP) Assay

The FRAP working solution was freshly prepared by mixing 0.3 M acetate buffer (pH 3.6), 0.01 M TPTZ in 0.04 M HCl, and 0.02 M FeCl_3_·6H_2_O in a 10:1:1 (*v*/*v*/*v*) ratio, and kept protected from light. Sample amounts (5–30 mL OLE, 4–10 mg OLE@NZ, or 10–40 mg film) were added to a mixture of 2.25 mL FRAP working solution and 0.225 mL deionized water. The reaction mixture was vortexed and incubated at 37 °C for 30 min in darkness. Absorbance was recorded at 593 nm. A blank was prepared using FRAP working solution and deionized water without any sample. The percentage of FRAP reduction was calculated using Equation (3):

(3) $$\%\;scavenged\;FRAP\;at\;steady\;state\;=\;\frac{A_{0}^{593}-A_{sample}^{593}}{A_{0}^{593}}\times 100$$

Linear regression of reduction percentage versus sample amount was used to determine EC_50,FRAP_. Each measurement was performed in triplicate.

All determinations were carried out in triplicate.

### 4.10. Total Polyphenol Content (TPC)

The TPC of OLE solution, OLE@NZ nanohybrid, fortified NaCl/OLE@NZ and Gel/Gl/xOLE@NZ film was measured by using a SHIMADJU UV/VIS spectrophotometer (UV-1900, Kyoto, Japan) via the following methodology.

The total phenolic content of OLE, OLE@NZ nanohybrid, fortified NaCl/OLE@NZ, and Gel/Gl/xOLE@NZ films was quantified using the Folin–Ciocalteu method with a UV-Vis spectrophotometer (UV-1900, Shimadzu, Kyoto, Japan).

For OLE: A 0.2 mL aliquot of the extract was transferred into a 5 mL volumetric flask containing 2.5 mL distilled water and 0.25 mL Folin–Ciocalteu reagent. After 3 min, 0.5 mL of saturated sodium carbonate solution (30% *w*/*v*) was added to establish alkaline conditions. The mixture was then diluted to 5 mL with either distilled water (pH 7), 1 M citric acid (pH 3.6), or 0.1 M HCl (pH 1). Following a 2 h incubation in the dark at room temperature, absorbance was measured at 760 nm. Results are expressed as mg gallic acid equivalents (GAE) per 100 mL of extract. Each sample was analyzed in triplicate.

For solid samples (OLE@NZ, fortified salt, and films): Approximately 10 mg of each material was stirred with 10 mL of ethanol, then filtered through 0.45 μm syringe filters. A 0.20 mL portion of the resulting ethanolic extract was added to a 5 mL volumetric flask containing 2.5 mL distilled water and 0.25 mL Folin–Ciocalteu reagent. After 3 min, 0.5 mL of 30% Na_2_CO_3_ was added. The final volume was adjusted to 5 mL using the same pH-specific media described above. After 2 h of dark incubation at room temperature, absorbance was read at 760 nm. TPC values are reported as mg GAE per unit mass of sample (or per extract volume), based on triplicate analyses.

### 4.11. Statistical Analysis

All experiments were performed in triplicate unless otherwise stated (tensile tests: n = 5). Results are expressed as mean ± standard deviation (SD). Statistical analyses were carried out using SPSS software (version 29.0, IBM Corp., Armonk, NY, USA). One-way analysis of variance (ANOVA) followed by Tukey’s honest significant difference (HSD) post hoc test was used to compare means among multiple groups. For comparisons between only two groups, paired or unpaired Student’s *t*-tests were applied as appropriate. A significance level of *p* < 0.05 was considered statistically significant. Homogeneity of variances was verified using Levene’s test (*p* > 0.05 for all datasets). Linear regressions for EC_50_ determinations were performed using Microsoft Excel, and the coefficient of determination (R^2^) was calculated to assess goodness of fit. All data met the assumptions of normality (Shapiro–Wilk test, *p* > 0.05) before ANOVA.

## 5. Conclusions

This study successfully developed a green, rapid microwave-assisted extraction protocol for producing an oleuropein-rich polyphenolic extract from olive leaves, a widely available agricultural by-product. The extract contained 25.4% (*w*/*w*) oleuropein and exhibited potent antioxidant activity. For the first time, this extract was effectively encapsulated into edible natural zeolite (clinoptilolite) via a simple adsorption-lyophilization process, forming OLE@NZ nanohybrids with strong radical scavenging activity and a polyphenol loading of approximately 30% *w*/*w*. The nanohybrid was then incorporated into two prototype food applications: a fortified salt (5% *w*/*w* OLE@NZ) and extruded gelatin-based active films (5, 10, and 15 wt.% OLE@NZ). The fortified salt fully preserved the antioxidant activity of the nanohybrid, while the gelatin films showed a concentration-dependent increase in antioxidant activity, with statistically significant enhancements of up to 14-fold compared to the blank film. Notably, the films exhibited a synergistic effect, with the same amount of nanohybrid being 5- to 7-fold more active when embedded in the gelatin matrix than in its pure form. The mechanical properties of the films remained acceptable, although high variability at 5% loading and embrittlement at 15% loading were observed. The total phenolic content of the films correlated linearly with the nanohybrid loading, confirming successful incorporation and extractability. However, further evaluations are required before commercial application, including long-term stability and shelf-life testing, antimicrobial assays, migration studies, real food packaging trials, and sensory analysis. Importantly, sensory evaluation (bitterness masking and overall acceptability) is a critical next step that was not performed in this study. Future work must include triangle tests with quinine references, hedonic scaling, and consumer acceptance panels [72,73,74]. Nevertheless, the present work demonstrates a complete circular economy pathway—from olive leaf valorization to functional food products—supporting the potential of OLE@NZ nanohybrids as sustainable, effective antioxidant additives for food preservation and nutrition.

## Figures and Tables

**Table 1:** List of identified bioactive compounds in the OLE.

Metabolite Number	Rt (min)	Calculated Mass (*m*/*z*)	Proposed Compound	Molecular Formula
**1**	16.4	315.4	Hydroxytyrosol glucoside isomer 1	C_14_H_20_O_8_
**2**	16.9	315.4	Hydroxytyrosol glucoside isomer 2	C_14_H_20_O_8_
**3**	17.1	389.4	Oleoside	C_16_H_22_O_11_
**4**	19.2	153.1	Hydroxytyrosol	C_8_H_10_O_3_
**5**	22.0	565.6	Elenolic acid diglucoside	C_23_H_33_O_16_
**6**	24.2	389.4	Secologanoside	C_16_H_23_O_11_
**7**	26.4	403.4	Oleoside methylester	C_17_H_24_O_11_
**8**	28.3	377.4	Decarboxyl enolic acid derivative	C_16_H_26_O_10_
**9**	30.6	403.4	Secoxyloganin	C_17_H_24_O_11_
**10**	34.7	447.4	Luteolin-7-O-glucoside	C_21_H_20_O_11_
**11**	40.6	539.5	Oleuropein	C_25_H_32_O_13_
**12**	42.7	539.5	Oleuropein isomer 1	C_25_H_32_O_13_
**13**	43.8	539.5	Oleuroside	C_25_H_32_O_13_
**14**	44.8	523.5	Ligstroside	C_25_H_32_O_12_
**15**	47.2	285.3	Luteolin	C_15_H_10_O_6_
**16**	51.0	269.4	Apigenin	C_15_H_10_O_5_

**Table 2:** Antioxidant activity (EC_50_) and total phenolic content (TPC) of the aqueous OLE. EC_50_ values are mean ± SD (*n* = 3). Different superscript letters indicate significant differences among assays (*p* < 0.05, one-way ANOVA followed by Tukey’s post hoc test). TPC is expressed as mg gallic acid equivalents per 100 mL of extract.

Parameter	Value
EC_50,DPPH_ (mL)	18.29 ± 0.44 ^a^
EC_50,ABTS_ (mL)	12.52 ± 0.41 ^c^
EC_50,FRAP_ (mL)	14.92 ± 0.40 ^b^
Total phenolic content (mg GAE/100 mL)	781.0 ± 11.3

**Table 3:** Antioxidant activity (EC_50_) and total phenolic content (TPC) of the OLE@NZ nanohybrid. EC_50_ values are mean ± SD (*n* = 3). Different superscript letters indicate significant differences among assays (*p* < 0.05, one-way ANOVA followed by Tukey’s post hoc test). TPC is expressed as mg gallic acid equivalents per liter of ethanolic extract.

Parameter	Value
EC_50,DPPH_ (mg/mL)	2.74 ± 0.44 ^b^
EC_50,ABTS_ (mg/mL)	1.87 ± 0.06 ^a^
EC_50,FRAP_ (mg/mL)	2.24 ± 0.07 ^a,b^
Total phenolic content (mg GAE/g)	425.69 ± 19.65

**Table 4:** Antioxidant activity (EC_50_) and total phenolic content (TPC) of the fortified salt NaCl/OLE@NZ (5% *w*/*w* OLE@NZ). EC_50_ values are mean ± SD (*n* = 3). Different superscript letters indicate significant differences among assays (*p* < 0.05, one-way ANOVA followed by Tukey’s post hoc test). TPC is expressed as mg gallic acid equivalents per liter of ethanolic extract.

Parameter	Value
EC_50,DPPH_ (mg salt)	50.82 ± 2.98 ^c^
EC_50,ABTS_ (mg salt)	34.68 ± 2.03 ^a^
EC_50,FRAP_ (mg salt)	41.54 ± 2.43 ^b^
Total phenolic content (mg GAE/g)	23.90 ± 5.04

**Table 5:** Tensile properties of Gel/Gl/OLE and Gel/Gl/xOLE@NZ films (mean ± SD, n = 5). Different superscript letters (a, b, c, d) within the same column indicate statistically significant differences (one-way ANOVA followed by Tukey’s HSD post hoc test, *p* < 0.05).

Sample	Elastic Modulus (MPa)	σ_uts_ (MPa)	Elongation at Break (%)
Gel/Gl/OLE (0%)	800.0 ± 32 ^a^	20.0 ± 1.2 ^a^	4.00 ± 0.16 ^a^
Gel/Gl/5OLE@NZ	1100.0 ± 44 ^b^	30.0 ± 1.5 ^b^	3.60 ± 0.14 ^b^
Gel/Gl/10OLE@NZ	1250.0 ± 50 ^c^	32.0 ± 1.6 ^c^	3.00 ± 0.12 ^c^
Gel/Gl/15OLE@NZ	1350.0 ± 54 ^d^	28.0 ± 1.4 ^d^	2.40 ± 0.10 ^d^

Standard deviations represent ~4% of the mean for modulus, ~5% for strength, and ~4% for elongation, consistent with typical variability in extruded biopolymer films.

**Table 6:** Antioxidant activity (EC_50_) of Gel/Gl/OLE and Gel/Gl/xOLE@NZ films (mean ± SD, *n* = 3). For each assay, different superscript letters indicate significant differences among film formulations (one-way ANOVA followed by Tukey’s post hoc test, *p* < 0.05). EC_50_ values are expressed as mg of film.

Sample	EC_50,DPPH_ (mg)	EC_50,ABTS_ (mg)	EC_50,FRAP_ (mg)
Gel/Gl/OLE (0%)	34.42 ± 9.94 ^a^	23.49 ± 6.78 ^a^	28.14 ± 8.12 ^a^
Gel/Gl/5OLE@NZ	8.65 ± 0.55 ^b^	5.90 ± 0.38 ^b^	7.07 ± 0.45 ^b^
Gel/Gl/10OLE@NZ	5.00 ± 0.30 ^c^	3.41 ± 0.20 ^c^	4.09 ± 0.25 ^c^
Gel/Gl/15OLE@NZ	2.50 ± 0.30 ^d^	1.71 ± 0.20 ^d^	2.04 ± 0.25 ^d^

**Table 7:** Total phenolic content (TPC) of Gel/Gl/OLE and Gel/Gl/xOLE@NZ films (mean ± SD, n = 3). Different superscript letters indicate significant differences among film formulations (one-way ANOVA followed by Tukey’s post hoc test, *p* < 0.05). TPC is expressed as mg gallic acid equivalents per gram of film (mg GAE/g).

Sample	TPC (mg GAE/g Film)
Gel/Gl/OLE (0%)	2.00 ± 0.17 ^a^
Gel/Gl/5OLE@NZ	14.40 ± 0.85 ^b^
Gel/Gl/10OLE@NZ	28.77 ± 1.16 ^c^
Gel/Gl/15OLE@NZ	36.97 ± 1.73 ^d^

**Table 8:** HPLC gradient elution program for the separation of phenolic compounds in OLE.

Time (min)	A (%)	B (%)
0.0	96.0	4.0
40.0	50.0	50.0
45.0	40.0	60.0
60.0	0.0	100.0
70.0	0.0	100.0
72.0	96.0	4.0
82.0	96.0	4.0

## Data Availability

The datasets generated for this study are available on request to the corresponding author.

## Supplementary Materials

The following supporting information can be downloaded at: https://www.mdpi.com/article/10.3390/molecules31111833/s1, Table S1: Individual EC_50_ values and linear regression parameters for OLE (DPPH, ABTS, FRAP); Table S2: Individual TPC values for OLE; Table S3: Individual EC_50_ values and linear regression parameters for OLE@NZ nanohybrid (DPPH, ABTS, FRAP); Table S4: Individual TPC values for OLE@NZ nanohybrid; Table S5: Individual EC_50_ values and linear regression parameters for fortified salt NaCl/OLE@NZ (DPPH, ABTS, FRAP); Table S6: Individual TPC values for fortified salt NaCl/OLE@NZ; Table S7: Individual tensile properties of Gel/Gl/OLE and Gel/Gl/xOLE@NZ films; Table S8: Individual EC_50_ values and linear regression equations for DPPH, ABTS, and FRAP assays of Gel/Gl/OLE and Gel/Gl/xOLE@NZ films; Table S9: Individual TPC values for Gel/Gl/OLE and Gel/Gl/xOLE@NZ films; Figure S1. Linear correlation between OLE@NZ nanohybrid loading (wt.%) and total phenolic content (TPC) of Gel/Gl/xOLE@NZ films. Data points represent mean ± standard deviation (n = 3). The solid line represents the linear regression fit (TPC = 2.33 × loading + 2.00, R^2^ = 0.995), indicating a strong proportional relationship between nanohybrid content and extractable polyphenols in the gelatin matrix.
