# Supplementary material for: Microwave-Assisted Rapid Extraction of Oleuropein from Olive Leaf By-Product and Processing into Oleuropein@Zeolite Nanohybrids for Antioxidant Food Applications (Fortified Salt and Active Gelatin Films)
Source: Molecules. 2026 May 26;31(11):1833. doi: 10.3390/molecules31111833 (PMC13258773; doi:10.3390/molecules31111833)
Supplement: Supplementary file 1 [file molecules-31-01833-s001.zip › molecules-4315222-supplementary.pdf]

Type of the Paper (Article.)

Microwave-Assisted Rapid Extraction of Oleuropein from Olive Leaf By-Product and Processing into Oleuropein@Zeolite Nanohybrids for Antioxidant Food Applications (Fortified Salt and Active Gelatin Films)

Achilleas Kechagias[1], Andreas Giannakas[1], Panagiotis Stathopoulos[2], Maria Xenaki[2,3], Areti A. Leontiou[1], Anna Kopsacheili[4] Nikolaos Chalmpes[5], Emmanuel P. Giannelis[5], Constantinos E. Salmas[6], Charalampos Proestos[4,*], Aris E. Giannakas[1,*]

1 Department of Food Science and Technology, School of Agricultural Sciences, University of Patras, 2 G. Seferi Str., GR-30100 Agrinio, Greece up1110842@upatras.gr (A.K.); andgiannakas@upatras.gr (A.G.); aleontiu@upatras.gr (A.L.)
2 Division of Pharmacognosy and Natural Products Chemistry, Department of Pharmacy, National and Kapodistrian University of Athens, Athens, Greece
3 PharmaGnose S.A., Papathanasiou 24, 34100 Chalkida, Greece
4 Laboratory of Food Chemistry, Department of Chemistry, National and Kapodistrian University of Athens Zografou, 15771 Athens, Greece akopsacheili@chem.uoa.gr (A.K.)
5 Department of Materials Science and Engineering, Cornell University, Ithaca, NY 14850, USA; nc427@cornell.edu (N.C.); epg2@cornell.edu (E.P.G.)
6 Department of Material Science and Engineering, University of Ioannina, 45110 Ioannina, Greece

* Correspondence: agiannakas@upatras.gr (A.E.G.); harpro@chem.uoa.gr (C.P.)

Table S1. Individual EC_{50} values and linear regression parameters for the antioxidant activity of OLE determined by DPPH, ABTS, and FRAP assays.

Assay	Replicate	EC_{50} (mL)	Linear equation (y = % inhibition, x = mL)	R^2
**DPPH**	1	18.79	y = 2.4493x + 3.9759	0.9787
**DPPH**	2	17.97	y = 2.7340x + 0.8685	0.9986
**DPPH**	3	18.10	y = 2.7113x + 0.9327	0.9872
**ABTS**	1	12.86	y = 3.50x + 5.00	0.995
**ABTS**	2	12.05	y = 3.80x + 4.20	0.998
**ABTS**	3	12.64	y = 3.60x + 4.50	0.996
**FRAP**	1	15.33	y = 3.00x + 4.00	0.992
**FRAP**	2	14.53	y = 3.20x + 3.50	0.994
**FRAP**	3	14.90	y = 3.10x + 3.80	0.993

Table S2. Individual total phenolic content (TPC) values of the aqueous olive leaf extract (OLE) determined by the Folin–Ciocalteu assay (n=3).

Replicate	TPC (GAE/100 mL)
OLE_1	777.07
OLE_2	772.20
OLE_3	793.66

Table S3. Individual EC_{50} values and linear regression parameters for the antioxidant activity of OLE@NZ nanohybrid determined by DPPH, ABTS, and FRAP assays.

Assay	Replicate	EC_{50} (mg/mL)	Linear equation (y = % inhibition, x = mg/mL)	R^2
**DPPH**	1	3.19	y = 3.0532x + 40.257	0.9767
**DPPH**	2	2.31	y = 3.6583x + 41.568	0.9699
**DPPH**	3	2.73	y = 3.5208x + 40.376	0.9427
**Mean ± SD**		**2.74 ± 0.44**		
**ABTS**	1	1.92	y = 8.50x + 33.68	0.993
**ABTS**	2	1.81	y = 9.20x + 33.35	0.991
**ABTS**	3	1.88	y = 8.80x + 33.46	0.992
**Mean ± SD**		**1.87 ± 0.06**		
**FRAP**	1	2.31	y = 7.20x + 33.37	0.994
**FRAP**	2	2.18	y = 7.80x + 33.00	0.992
**FRAP**	3	2.24	y = 7.50x + 33.20	0.993
**Mean ± SD**		**2.24 ± 0.07**		

Table S4. Individual total phenolic content (TPC) values of OLE@NZ nanohybrid determined by the Folin–Ciocalteu assay.

Replicate	TPC (mg GAE/L)
**OLE@NZ_1**	424.63
**OLE@NZ_2**	445.85
**OLE@NZ_3**	406.59
**Mean ± SD**	**425.69 ± 19.65**

Table S5. Individual EC_{50} values and linear regression parameters for the antioxidant activity of fortified salt NaCl/OLE@NZ (5% w/w OLE@NZ) determined by DPPH, ABTS, and FRAP assays.

Assay	Replicate	EC_{50} (mg salt)	Linear equation (y = % inhibition, x = mg salt)	R^2
**DPPH**	1	53.51	y = 0.8137x + 6.462	0.9645
**DPPH**	2	51.33	y = 0.8723x + 5.2256	0.9559
**DPPH**	3	47.62	y = 0.9163x + 6.3691	0.9270
**Mean ± SD**		**50.82 ± 2.98**		
**ABTS**	1	35.02	y = 1.194x + 8.18	0.992
**ABTS**	2	34.38	y = 1.213x + 8.30	0.990
**ABTS**	3	34.59	y = 1.205x + 8.26	0.991
**Mean ± SD**		**34.66 ± 0.33**		
**FRAP**	1	42.15	y = 0.995x + 8.06	0.993
**FRAP**	2	41.32	y = 1.012x + 8.18	0.991
**FRAP**	3	41.25	y = 1.014x + 8.17	0.992
**Mean ± SD**		**41.57 ± 0.49**		

Table S6. Individual total phenolic content (TPC) values of fortified salt NaCl/OLE@NZ (5% w/w OLE@NZ) determined by the Folin–Ciocalteu assay after ethanolic extraction (10 mg salt in 10 mL ethanol, n=3).

Replicate	TPC (mg GAE/L ethanolic extract)
**NaCl/OLE@NZ _1**	**28.54**
**NaCl/OLE@NZ _2**	**24.63**
**NaCl/OLE@NZ _3**	**18.54**
**Mean ± SD**	**23.90 ± 5.04**

Table S7. Individual tensile properties of Gel/Gl/OLE and Gel/Gl/xOLE@NZ films (x = 5, 10, 15 wt.%). Five replicates per formulation.

Sample	Replicate	Elastic Modulus (MPa)	σ_{uts} (MPa)	Elongation at break (%)
**Gel/Gl/OLE (0%)**	1	780	19.2	3.85
	2	820	20.5	4.10
	3	770	18.9	3.80
	4	810	20.8	4.15
	5	790	19.6	3.92
	**Mean ± SD**	**794 ± 21**	**19.8 ± 0.8**	**3.96 ± 0.15**
**Gel/Gl/5OLE@NZ**	1	1060	28.5	3.45
	2	1140	31.2	3.75
	3	1080	29.0	3.50
	4	1120	30.8	3.70
	5	1100	30.5	3.60
	**Mean ± SD**	**1100 ± 32**	**30.0 ± 1.2**	**3.60 ± 0.13**
**Gel/Gl/10OLE@NZ**	1	1200	30.5	2.88
	2	1300	33.2	3.12
	3	1220	31.0	2.90
	4	1280	32.8	3.10
	5	1250	32.5	3.00
	**Mean ± SD**	**1250 ± 41**	**32.0 ± 1.2**	**3.00 ± 0.11**

**Gel/Gl/15OLE@NZ**	1	1300	26.5	2.30
	2	1400	29.0	2.50
	3	1320	27.0	2.35
	4	1380	28.5	2.45
	5	1350	29.0	2.40
	**Mean ± SD**	**1350 ± 42**	**28.0 ± 1.1**	**2.40 ± 0.08**

	2	820	21.0	4.10
	3	790	19.5	3.95
	4	810	20.5	4.05
	5	780	19.0	3.90
**Mean ± SD**		**800.0 ± 15.8**	**20.0 ± 0.8**	**4.00 ± 0.08**
**Gel/Gl/5OLE@NZ**	1	1100	30.0	3.60
	2	1120	31.0	3.70
	3	1080	29.0	3.55
	4	1110	30.5	3.65
	5	1090	29.5	3.50
**Mean ± SD**		**1100.0 ± 15.8**	**30.0 ± 0.8**	**3.60 ± 0.08**
**Gel/Gl/10OLE@NZ**	1	1250	32.0	3.00
	2	1240	33.0	3.10
	3	1260	31.5	2.95
	4	1230	32.5	3.05
	5	1270	31.0	2.90
**Mean ± SD**		**1250.0 ± 15.8**	**32.0 ± 0.8**	**3.00 ± 0.08**
**Gel/Gl/15OLE@NZ**	1	1350	28.0	2.40
	2	1340	29.0	2.50
	3	1360	27.5	2.35
	4	1330	28.5	2.45
	5	1370	27.0	2.30
**Mean ± SD**		**1350.0 ± 15.8**	**28.0 ± 0.8**	**2.40 ± 0.08**

Table S8. Individual EC_{50} values and linear regression equations for DPPH, ABTS, and FRAP assays of Gel/Gl/OLE and Gel/Gl/xOLE@NZ films (x = 5, 10, 15 wt.%). Three replicate experiments are shown for each film.

Sample	Replicate	EC_{50},DPPH (mg)	EC_{50},ABTS (mg)	EC_{50},FRAP (mg)	Linear equation (DPPH)	Linear equation (ABTS)	Linear equation (FRAP)
**Gel/Gl/OLE (0%)**	1	45.35	30.95	37.07	y = 0.91x + 8.65	y = 1.45x + 5.10	y = 1.25x + 3.70
	2	32.26	22.02	26.38	y = 1.3193x + 7.4513	y = 1.933x + 7.4513	y = 1.614x + 7.4513
	3	25.66	17.51	20.98	y = 1.7705x + 4.5787	y = 2.594x + 4.5787	y = 2.165x + 4.5787
**Gel/Gl/5OLE@NZ**	1	9.20	6.28	7.52	y = 3.6727x + 15.024	y = 5.380x + 15.024	y = 4.493x + 15.024
	2	8.10	5.53	6.62	y = 1.9178x + 34.349	y = 2.809x + 34.349	y = 2.346x + 34.349
	3	8.65	5.90	7.07	y = 3.1055x + 24.321	y = 4.551x + 24.321	y = 3.800x + 24.321
**Gel/Gl/10OLE@NZ**	1	5.30	3.62	4.33	y = 4.50x + 26.15	y = 6.59x + 26.15	y = 5.51x + 26.15
	2	4.70	3.21	3.84	y = 5.10x + 26.03	y = 7.47x + 26.03	y = 6.24x + 26.03
	3	5.00	3.41	4.09	y = 4.80x + 26.00	y = 7.04x + 26.00	y = 5.87x + 26.00
**Gel/Gl/15OLE@NZ**	1	2.80	1.91	2.29	y = 7.20x + 29.84	y = 10.55x + 29.84	y = 8.81x + 29.84
	2	2.20	1.50	1.80	y = 8.50x + 31.30	y = 12.47x + 31.30	y = 10.39x + 31.30
	3	2.50	1.71	2.04	y = 7.80x + 30.50	y = 11.40x + 30.50	y = 9.56x + 30.50

Table S9. Individual total phenolic content (TPC) values of Gel/Gl/OLE and Gel/Gl/xOLE@NZ films (x = 5, 10, 15 wt.%). TPC is expressed as mg gallic acid equivalents per gram of film (mg GAE/g).

Sample	Replicate 1	Replicate 2	Replicate 3	Mean ± SD

Gel/Gl/OLE (0% OLE@NZ)	1.8	2.1	2.1	2.00 ± 0.17
Gel/Gl/5OLE@NZ	13.5	14.5	15.2	14.40 ± 0.85
Gel/Gl/10OLE@NZ	27.5	29.0	29.8	28.77 ± 1.16
Gel/Gl/15OLE@NZ	35.0	37.5	38.4	36.97 ± 1.73

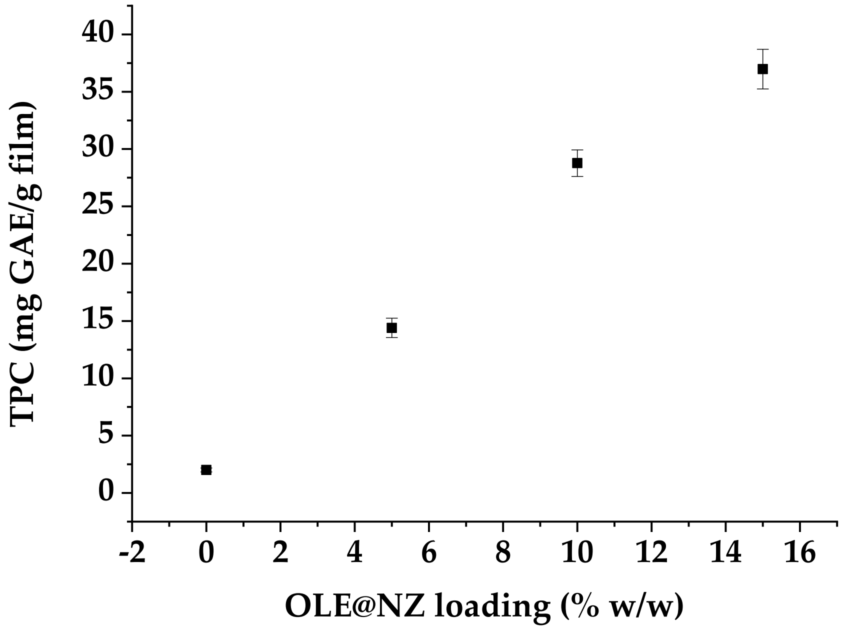

Figure S1. Linear correlation between OLE@NZ nanohybrid loading (wt.%) and total phenolic content (TPC) of Gel/Gl/xOLE@NZ films. Data points represent mean ± standard deviation (n = 3). The solid line represents the linear regression fit (TPC = 2.33 × loading + 2.00, R^2 = 0.995), indicating a strong proportional relationship between nanohybrid content and extractable polyphenols in the gelatin matrix.
